# Phytochemical Composition and Antioxidant, Anti-Acetylcholinesterase, and Anti-α-Glucosidase Activity of *Thymus carnosus* Extracts: A Three-Year Study on the Impact of Annual Variation and Geographic Location

**DOI:** 10.3390/antiox12030668

**Published:** 2023-03-08

**Authors:** Carlos Martins-Gomes, Jan Steck, Judith Keller, Mirko Bunzel, João A. Santos, Fernando M. Nunes, Amélia M. Silva

**Affiliations:** 1 Centre for Research and Technology of Agro-Environmental and Biological Sciences (CITAB), University of Trás-os-Montes and Alto Douro (UTAD), Quinta de Prados, 5001-801 Vila Real, Portugal; camgomes@utad.pt (C.M.-G.); jsantos@utad.pt (J.A.S.); 2 Chemistry Research Centre—Vila Real (CQ-VR), UTAD, Quinta de Prados, 5001-801 Vila Real, Portugal; fnunes@utad.pt; 3 Department of Food Chemistry and Phytochemistry, Institute of Applied Biosciences, Karlsruhe Institute of Technology (KIT), Adenauerring 20a, 76131 Karlsruhe, Germanyjudith.keller@kit.edu (J.K.); mirko.bunzel@kit.edu (M.B.); 4 Department of Physics, School of Sciences and Technology, UTAD, Quinta de Prados, 5001-801 Vila Real, Portugal; 5 Department of Chemistry, School of Life and Environmental Sciences (ECVA), UTAD, Quinta de Prados, 5001-801 Vila Real, Portugal; 6 Department of Biology and Environment, ECVA, UTAD, Quinta de Prados, 5001-801 Vila Real, Portugal

**Keywords:** antioxidant activity, neuroprotective potential, anti-diabetic potential, *Thymus carnosus* Boiss., phytochemical profile, climate variability

## Abstract

*Thymus carnosus* Boiss. is a near-threatened species, and, as for many species, its potential for medicinal purposes may be lost if measures towards plant protection are not taken. A way of preserving these species is to increase knowledge about their medicinal properties and economic potential. Thus, with the objective of studying the potentiality of introducing *T. carnosus* as a crop, the stability of the phytochemical profile of *T. carnosus* was studied during a period of three years by comparing the phytochemical profile of extracts obtained from plants harvested in two different edaphoclimatic locations, as well as by comparing the respective bioactivities, namely, antioxidant, antidiabetic, antiaging, and neuroprotective activities. It was reported, for the first time, the effect of annual variation and geographic location in the phytochemical composition of aqueous decoction and hydroethanolic extracts of *T. carnosus*. In addition, the presence of two salvianolic acid B/E isomers in *T. carnosus* extracts is here described for the first time. Despite the variations in phytochemical composition, according to harvesting location or year, *T. carnosus* extracts maintain high antioxidant activity, assessed by their capacity to scavenge ABTS^•+^, ^•^OH , NO^•^, O_2_^•−^ radicals, as well as to prevent β-carotene bleaching. All extracts presented significant potential to inhibit acetylcholinesterase (AChE), tyrosinase, and α-glucosidase, denoting neuroprotective, anti-aging, and anti-diabetic potential. In conclusion, the vegetative stage and location of harvest are key factors to obtain the maximum potential of this species, namely, a phytochemical profile with health benefit bioactivities.

## 1. Introduction

Nowadays, the food, pharmaceutical, and cosmetic industries have a growing and constant demand for new products and ingredients with health promoting effects, with an emphasis on natural products. In addition to the general low cost and safe use, the consumption of natural products attracts consumers [1]. Medicinal and aromatic plants are within the natural products with higher potential applications in these industries, where plants from *Thymus* L., *Mentha* L., *Salvia* L., and other genera belonging to the Lamiaceae family are already used as condiments and preservatives in the food sector [2,3,4,5,6], as well ingredients in functional beverages [7] and other products.

Nevertheless, not all species have yet been approached for these purposes, and due to anthropogenic action and loss of natural habitats, a large number of plant species, which currently do not present industrial or commercial use, have been listed in the IUCN’s (International Union for Conservation of Nature) Red List of Threatened Species [8], some of them belonging to the *Thymus* genus. This is the case for *Thymus carnosus* Boiss., a near-threatened species commonly known as beach thyme, and it is endemic to the Iberian Peninsula, growing mainly on Portugal’s south and southwest shores [9,10].

In the same way as reported for other *Thymus* species listed in IUCN’s Red List, such as *Thymus albicans* (Hoffmanns. and Link), which is listed as vulnerable [11,12], a large number of these species are only now beginning to be characterized regarding their phytochemical composition, bioactivities, and potential use for human health benefit. Through the study of its health-promoting effects, it is expected to raise awareness towards a sustainable crop aiming at later industrial applications, as well as the maintenance of biodiversity. According to the IUCN’s latest Red List report (9 December 2022), among the 60,470 flowering plant species assessed since 1996, 24,000 were listed as threatened (sum of critically endangered, endangered, or vulnerable species) [13]. Bernardini, et al. 2018 [14] reported, in 2017, that only approximately 60,000 plant species were screened for pharmaceutical uses, from which 135 pharmaceutical products originated [14]. Given that the total number of flowering plant species described by IUCN ascends to 369,000 species, it is clear that the knowledge regarding the health-promoting activities of flowering plans is still far from its maximum potential. Even more, considering species such as *T. carnosus*, listed in IUCN’s Red List of Threatened Species, there is a risk of losing the knowledge and potential applications.

By increasing the potential applications of *T. carnosus*, and its value to the industry, it is expected that actions towards a sustainable crop occur, thus benefiting the maintenance of biodiversity, as well as also the diversity of pharmaceutical options. As limitations to increase the interest in poorly studied species, it can be pointed out that: (1) the absence of a complete phytochemical composition, as well as the correlation of phenolic compounds with various potential bioactivities; (2) knowledge regarding its safety profile, to be further included in human diet, as well as health-promoting effects; and (3) its suitability to be adapted to agricultural production in order to be proposed as a sustainable source of medicinal effects and bioactive compounds.

Aiming at the preservation of *T. carnosus*, regarding the points described above, our group has recently described, for the first time, the phytochemical composition of aqueous decoction and hydroethanolic extracts of this species. HPLC-DAD-ESI-MS^n^ analysis of these extracts revealed a unique phytochemical composition, mainly rich in phenolic acids, such as rosmarinic acid (RA), salvianolic acids A (SAA), and K (SAK), as well as a novel salvianolic acid A isomer (SAA iso), which are present in high quantities, as well as glycosidic derivatives of luteolin [9]. In addition, high quantities of oleanolic (OA) and ursolic (UA) acids were found in hydroethanolic extracts [9]. These results highlighted these extracts as a source of phytochemicals of high pharmaceutical value, which required posterior validation. Thus, using human cell culture models of colorectal carcinoma (Caco-2), hepatocarcinoma (HepG2), breast adenocarcinoma (MCF-7), and mammary gland ductal carcinoma (BT-474), both aqueous and hydroethanolic extracts were shown to induce anti-proliferative effect against these tumoral cell lines, the hydroethanolic extract being the one with higher effect [9,15]. In Caco-2 cells, the anti-proliferative/cytotoxic effect was correlated with apoptosis induction, cell cycle arrest, and morphological changes [15]. In addition, both extracts revealed anti-inflammatory potential, higher for the aqueous extracts, observed during the reduction of nitric oxide production in a lipopolysaccharide-stimulated macrophage cell model [9]. However, the sustainable crop requires the knowledge of the stability of *T. carnosus* phytochemical profile and the ability to expand its habitat, which are dependent on other factors, such as the geographic location and edaphoclimatic conditions, which may induce changes in the phytochemical composition.

In the *Thymus* genus, most studies analysing the effects of edaphoclimatic conditions, inter-year climate changes, and vegetative stage are mainly towards the composition of essential oils, being reported that these factors modulate the essential oils’ phytochemical composition, and therefore their bioactivities, as described for *Thymus vulgaris* L. [16], *Thymus pulegioides* L. [17], *Thymus pallescens* Noë. [18], or *Thymus hyemalis* Lange [19]. For *Thymus vulgaris* [16], in addition to the phytochemical composition, a change in antioxidant and antibacterial activities was also observed. Regarding thyme extracts, using *Thymus longicaulis* C. Presl collected in different seasons, it was observed that its hydro-methanolic (1:1; % *v*/*v*) extract’s polyphenolic composition presented significant variances [20]. The main compound, RA, varied from 12.97 to 3029.56 µg/mL (in quercetin equivalents) in plants harvested from July to October of the same year. However, other phenolic acids also showed variations with the time of harvest and, for example, salvianolic acid K showed a concentration variation inverse to that of RA, and this was also observed for other phenolic acids. The seasonal variance effect was also clearly observed in the tested bioactivities, with significant differences for anti-inflammatory, anti-proliferative, and antioxidant activities [20]. The variation of pentacyclic triterpenoids concentration, such as OA and UA (compounds present in high amounts in *T. carnosus* HE extract [9]), induced by the vegetative stage, has been described in various thyme species. Analysed in methanolic extracts, small variations in OA and UA content through the various vegetative phases was reported in *Thymus praecox* ssp. *arcticus* Opiz. extracts, while, in other species, such as *Thymus pulegioides* at the end of vegetative stage OA and UA content, was 2.2 and 2.98 times higher than the value at fruit maturation stage [21]. Interestingly, the stage with higher content in each pentacyclic triterpenoids is dependent on the species under study [21], highlighting the need to understand the best conditions for each species.

Therefore, considering *T. carnosus* as a potential crop, the aim of this work was to study the stability of *T. carnosus* phytochemical profile over a period of three years to compare the phytochemical profile of extracts obtained from plants harvested in two different edaphoclimatic conditions, as well as to compare the respective bioactivities, namely, antioxidant, anti-diabetic, anti-aging, and neuroprotective activities. For this reason, in the present study, aerial parts of *T. carnosus* were collected in November, a post-flowering stage, in which the phytochemical profile of the plant may reflect the environmental stresses experienced in the previous months. Aerial parts were collected both at its natural habitat, where it grows as an endemic wild plant, as well as at UTAD’s botanical garden, where the plant has adapted to a different climatic condition. The first location generally presents higher average temperatures in the last trimester of the year when compared to UTAD’s botanical garden. In addition, precipitation is usually higher in northern Portugal, which may also induce variations in the phytochemical profile. The composition of aqueous and hydroethanolic extracts in phenolic and terpenoid compounds was assessed by chromatographic methodologies and correlated to its potential as an antioxidant, anti-diabetic, anti-aging, and/or neuroprotective agent.

## 2. Materials and Methods

### 2.1. Standards and Reagents

Commercial standards used for HPLC identification and quantification were purchased from Sigma-Aldrich/Merck (Algés, Portugal), Extrasynthese^®^ (Genay, France), and Santa Cruz Biotechnology Inc. (Frilabo, Porto, Portugal). All solvents used were HPLC or PA grade and were obtained from Sigma-Aldrich/Merck (Algés, Portugal). Folin-Ciocalteu’s reagent, sodium carbonate, sodium molybdate, aluminium chloride (III), sodium nitrite, (±)-6-hydroxy-2,5,7,8-tetramethylchromane-2-carboxylic acid (Trolox), 2,2-azino-bis (3-ethylbenzothiazoline-6-sulfonic acid) diammonium salt (ABTS), potassium persulfate, sodium nitroprusside, sulfanilamide, *N*-(1-naphthyl)ethylenediamine dihydrochloride, β-carotene, linoleic acid, xanthine oxidase, hypoxanthine, nitro blue tetrazolium, ascorbic acid, ethylenediaminetetraacetic acid (EDTA), hydrogen peroxide (30% solution), trichloroacetic acid (TCA), thiobarbituric acid (TBA), and 2-deoxy-D-ribose were used. Enzymes and reagents for enzymatic assays were purchased from Sigma-Aldrich/Merck (Algés, Portugal). Other salts and reagents not mentioned above were obtained from Sigma-Aldrich/Merck (Algés, Portugal).

### 2.2. Plant Material

**Figure 1:**
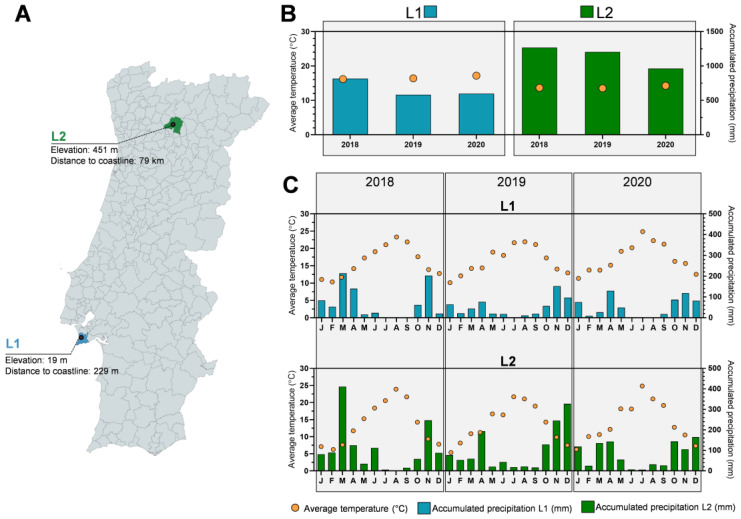
Identification of harvesting locations and climatic conditions in which the *Thymus carnosus* used in this work grew. Geographic location of *T. carnosus* harvest points, elevation from sea level and distance to coastline, nearest location in straight-line distance (**A**), and annual (**B**) and mensal (**C**) variation of average temperature (in °C; yellow dots) and accumulated precipitation for 2018–2020 period in L1 (blue bars) and L2 (green bars).

Aerial parts (constituted by leaves and stems) of *T. carnosus* Boiss. were collected in Arrábida National Park (coordinates: latitude 38.492637°/longitude −9.181475°; Sesimbra, Setúbal, Portugal), further identified as location one (L1) and in the Botanical Garden of the University of Trás-os-Montes e Alto Douro (coordinates: latitude 41.287538°/longitude −7.740203°; UTAD), and further identified as location two (L2), in November of 2018, 2019, and 2020. L1 harvest was dependent on authorization granted by the Portuguese Institute for Nature Conservation and Forests (ICNF) (License no. 867/2018/RECOLHA; 868/2018/RECOLHA; 723/2019/RECOLHA; 723/2019/RECOLHA; 198/2020/RECOLHA; 199/2020/RECOLHA). A portion of the plant material (containing leaves and stems) harvested in L1-2018 was used for authentication by the Botanical Garden office at the University of Trás-os-Montes and Alto Douro (UTAD, Vila Real, Portugal), originating the voucher specimen nº HVR22496, and the following harvests were performed in the same exact location. The existing specimen in UTAD’s botanical garden had been previously identified with the voucher nº HVR21093. L1 and L2 localization, climate parameters (average temperature (°C), and accumulated precipitation (mm)) in the 2018–2020 period, as well as relevant geographical parameters, are schematized in Figure 1. L1′s data were obtained through a dataset of Portugal’s weather conditions developed and described by Fonseca and Santos 2018 [22]. For L2, the data were retrieved from a weather station located at UTAD. After each harvest, the plant material was rinsed with distilled water, weighted, frozen, and lyophilized (Dura Dry TM μP freeze-drier; −45 °C and 250 mTorr). After this step, the plant material was ground and stored in a cool and dry place, protected from light, until further extraction and analysis.

### 2.3. Preparation of Extracts

Aqueous and hydroethanolic extracts were obtained as described by Martins-Gomes et al. (2018) [9], using aqueous decoction extraction (AD) and exhaustive hydroethanolic (HE) extraction procedures, respectively. Briefly, 0.5 g of lyophilized, ground plant material were used for both methods. To obtained AD extracts, 150 mL of distilled water were added to the plant material, followed by heating to 100 °C, where it was maintained for 20 min, under agitation. After this period, the mixture was allowed to cool down, to room temperature, and then was filtered. HE exhaustive extraction comprised a three-step sequential extraction method of the plant material with 50 mL of an ethanol:water solution (80:20, % *v*/*v*), each of the steps being under agitation (orbital shaker; 150 rpm) for one hour and then centrifuged (7000 rpm, Sigma Centrifuges 3–30 K, St. Louis, MO, USA). The three supernatants were collected, combined, and filtered. Both extracts were filtered twice (Whatman nº 4 filter and fiberglass filter (1.2 µm; acquired from VWR International Ltd., Alfragide, Portugal)) and concentrated to 100 mL in a rotary evaporator (35 °C), the step in which the ethanol was removed from the HE extract [9]. These methodologies were repeated three times, and all extracts were frozen and lyophilized, followed by weighing for yield calculation and proper storing until further analysis.

### 2.4. Total Phenolic Compounds, Total Flavonoids and Ortho-Diphenols Content

Total phenolic compounds content (TPC), total flavonoid content (TFC), and *ortho*-diphenol content (ODC) were quantified using colorimetric reactions based on Folin-Ciocalteau reagent, molybdenum complexation, and aluminium complexation, respectively. All methodologies were performed as described by Taghouti, et al. 2020 [23]. TPC and ODC were expressed as caffeic acid equivalents (mg CA eq./g lyophilized plant or mg CA eq./g extract), and TFC was expressed as catechin equivalents (mg C eq/g lyophilized plant or mg C eq/g extract).

### 2.5. Phytochemical Composition Profiling and Quantification by HPLC-DAD and HPLC-ESI-MS^n^

Individual phenolic compounds, oleanolic acid, and ursolic acid identification and quantification were performed by RP-HPLC-DAD analysis using a Vanquish Core HPLC system (Thermo Fisher Scientific, Waltham, MA, USA) equipped with auto-sampler, pump, column compartment, and diode array detector. Chromatographic separation was performed using a C18 column (Merck Purospher^®^ STAR, Hibar^®^ C18; 250 mm × 4.6 mm; particle size 5 μm), with an injection volume of 100 μL, and the temperature kept at 40 °C, and the flow rate was 0.5 mL/min. 

The elution system used for phenolic compounds consisted of solvent A (0.1% formic acid prepared in ultra-pure distilled water, *v*/*v*) and solvent B (methanol) with the elution profile as follows: 0–15 min, 10–30% B (*v*/*v*); 15–60 min, 30–56% B; 60–65 min, 56–100% B; 65–66 min, 100–10% B; and 66–75 min, 10% B (equilibration). The total acquisition time was 65 min, and the total run time was 75 min.

Chromatographic separation of terpenoids was achieved using the same column, solvents, injection volume, flow rate, and temperature described above, using the following elution system: 0–45 min, 80–90% B; 45–54 min, 90% B; 54–55 min, 90–80% B; and 55–65 min, 80% B (equilibration). The total acquisition time was 55 min, and the total run time was 65 min.

UV/Vis detection was performed at 200–600 nm, being 280 nm and 325 nm, which were used for phenolic compound quantification, and 210 nm was used for terpenoid quantification. Chromeleon software (Version 7.3; Dionex, USA) was used for data acquisition, peak integration, and analysis.

RP-HPLC-ESI-MS^n^ analysis was performed for accurate phenolic compounds identification using a Thermo Scientific system equipped with a Finnigan Surveyor Plus auto-sampler, pump, LXQ Linear ion trap detector, and a photodiode array detector. The elution system, column, solvents, temperature, flow rate, injection volume, and detection parameters were performed as described by Martins-Gomes et al. (2018) [9].

The identification of individual phenolic compounds present in *T. carnosus* extracts was based on the data acquired from UV-VIS and mass spectrometry analysis, as well as retention time comparison with commercial standards and/or literature data. Oleanolic and ursolic components were identified only by HPLC-DAD by comparison to their respective commercial standards. Phytochemicals’ quantification was performed based on calibration curves of commercial standards, if available, or using the aglycones or standard compounds with structural similarity to commercial standards. Caffeic acid (CA; PubChem CID: 689043) was quantified as its respective standard. Luteolin and apigenin derivatives were quantified as luteolin-7*-O-*glucoside (L-7-G; PubChem CID: 5280637); quercetin derivatives were quantified as quercetin-3*-O-*glucoside (Pubchem CID 25203368); eriodyctiol derivatives were quantified as eriodyctiol-7*-O-*glucoside (Pubchem CID 13254473); RA, SAA iso, SAK, and salvianolic acid K isomer (SAK iso) were quantified as RA (PubChem CID: 5281792). 

### 2.6. In Vitro Antioxidant Activity Assessment

In vitro radical scavenging capacity of *T. carnosus* aqueous and hydroethanolic extracts was evaluated using ABTS (ABTS^•+^) and superoxide (O_2_^•−^) radicals scavenging and β-carotene bleaching assays. ABTS^•+^ scavenging assay was performed, as described by Taghouti et al. (2018) [24] and expressed as mmol Trolox equivalent/g dry plant. Trolox was also used as a positive control (IC_50_ = 0.24±0.01 mg/mL) 

Regarding O_2_^•−^ scavenging by *T. carnosus* extracts, 6.7 µL of extracts were added to 193.3 µL of the reaction mixture (174 µL of phosphate buffer (50 mM; pH 8), 12.86 µL of nitro blue tetrazolium solution (NBT; 4 mM) and 6.43 µL of hypoxanthine solution (4 mM)), and the mixture was incubated 2 min at 37 °C. The reaction was initiated with the addition of xanthine oxidase solution (20 µL at 0.04 U/mL; in 50 mM phosphate buffer (pH 8) supplemented with 500 µM EDTA). The absorbance was first measured immediately after enzyme addition (blank) at 570 nm (Multiskan EX microplate reader (MTX Labsystems; Bradenton, Florida, USA)). After a 20 min incubation at 37 °C, 20 µL of HCl (0.6 M) were added to stop the reaction, followed by a second absorbance measurement at 570 nm. Rosmarinic acid was used as a positive control (95.71 ± 8.55% inhibition at 120 µg/mL).

For β-carotene bleaching assay, the emulsion was prepared by adding 500 mg of between 20 to 250 µL of β-carotene solution (2 mg/mL solution; in chloroform) and was followed by mixing in a round-bottom evaporation flask [25]. After evaporating the solvent in a rotary evaporator (35 °C), 25 mg of linoleic acid and 50 mL of distilled water were added, in this order. To produce the emulsion, the mixture was then gently homogenized using the rotary evaporator (rotary motion with no vacuum) at room temperature. The assay was carried out in a 96-well microplate, in which 50 µL of the extracts were added to 250 µL of the emulsion, followed by blank measurement at 450 nm. After 2 h incubation at 50 °C, the microplates were placed over ice to stop the reaction, in the dark, for two minutes, followed by a second absorbance measurement. Hydroethanolic extracts were dissolved in 10% (*v*/*v*) DMSO solution, and then they were tested to assure no interference with the assay. Rosmarinic acid was used as a positive control (IC_50_ = 22.05 ± 1.02 µg/mL).

Hydroxyl (^•^OH) and nitric oxide (NO^•^) radicals scavenging assays were only performed for aqueous extract, due to ethanol interference, as the HE extracts are not fully water-soluble. Both assays were performed as described by Taghouti et al. (2020) [23]. With the exception of ABTS^•+^ scavenging assay (tested at 1 mg/mL), a range of concentrations of the extracts (0.1–1 mg/mL) was analyzed, and results are expressed as inhibition percentage and IC_50_, calculated according to equation 1. Distilled water was used as the negative control, and rosmarinic acid was used as a positive control for ^•^OH scavenging assay without EDTA (43.27 ± 3.50% inhibition at 45 µg/mL) and for the NO^•^ scavenging assay (44.43 ± 2.62% inhibition at 15 µg/mL).

(1) $$Inhibition\;\left(\%\right)=\frac{\mathrm{Blank}\;\mathrm{abs}-\mathrm{Sample}\;\mathrm{abs}\;}{\mathrm{Blank}\;\mathrm{abs}\;}\times 100$$

### 2.7. Enzymatic Inhibition Assays

*T. carnosus* aqueous decoction and hydroethanolic extracts were studied for their potential neuroprotective, anti-aging, and anti-diabetic activities. These bioactivities were evaluated based on the capacity to inhibit key enzymes of target metabolic pathways. Acetylcholinesterase (AChE) and tyrosinase inhibition were evaluated for neuroprotection, tyrosinase and elastase for anti-aging activity, and α-amylase and α-glucosidase for anti-diabetic activity.

All methodologies were performed using colorimetric assays, as described by Taghouti et al. (2018) [24]. All extracts were tested in a range of concentrations from 0.1 to 1 mg/mL. Hydroethanolic extract dilutions were prepared from a DMSO stock solution, and they never exceed 2.5% DMSO final concentration. A control was performed in all assays to exclude DMSO interference, and distilled water was used as control (blank). As positive controls, quercetin was used for AChE (48.61 ± 3.50% inhibition at 120 µg/mL) and elastase (51.20 ± 7.20% inhibition at 120 µg/mL), kojic acid for tyrosinase (97.04 ± 1.09% inhibition at 1 mg/mL), and acarbose for α-amylase (79.48 ± 3.62% inhibition at 1 mg/mL) and α-glucosidase (76.67 ± 1.33% inhibition at 1 mg/mL).

### 2.8. Statistical Analysis

The experimental assays were performed for all the extracts obtained in each extraction method, with three experimental repetitions for each extract. Analysis of variance (ANOVA), followed by Tukey’s multiple tests, were performed to analyze statistically significant differences. Correlations were evaluated using Pearson’s coefficient (significant if *p* < 0.05). The IC_50_ values were obtained from the dose–response assays described above and calculated, as described by Silva, et al. 2019 [26]. Principal component analysis (PCA) was used to evaluate inter-year variance of the individual phenolic components and performed as described by Ferreira, et al. 2020 [27]. The correlation between individual phytochemicals and antioxidant or enzymatic inhibition activities was performed through orthogonal partial least squares-discriminant analysis (OPLS-DA), as described by Martins, et al. 2022 [28]. Statistical analyses and graphic design were performed using Statistica (Version 14; TIBCO Software Inc., California, USA), SIMCA software (Version 14.1. Umetrics, Umea, Sweden), GraphPad Prism (Version 8; GraphPad Software Inc, California, USA), and Microsoft Office Excel (Microsoft Corporation, Washington, DC, USA).

## 3. Results and Discussion

Medicinal and aromatic plants’ phytochemical composition is known to present high heterogeneity, even in species belonging to same genus. An example is the *Thymus* genus, since several species, such as *T. pulegioides* [24], *Thymus zygis* Loefl. ex L. [29], *Thymus fragrantissimus* [30], *Thymus mastichina* L. [23], *Thymus × citriodorus* (Pers.) Schreb. [31], and *T. vulgaris* [31], were harvested in the same location, grown in the same conditions, and whose phytochemicals were extracted using the same methodologies, and the extracts presented different yields, phytochemical profiles, and bioactivities. In addition to inter-species genetic variations, factors, such the vegetative phase and edaphoclimatic conditions, play a critical role in phytochemical composition variation [27,32,33,34]. The latter is being widely discussed in light of climate changes induced by global warming. The effect of edaphoclimatic factors, such as temperature, precipitation or soil chemistry, and moisture, is well established as a determinant of secondary metabolite production [27,32,33,34].

Within the *Thymus* genus, several studies have been performed to evaluate these variations, *T. vulgaris* and its essential oils being the most frequently addressed, given its significant economic impact. Lemos et al. (2017) studied the seasonal variance of *T. vulgaris*’ essential oil from plants harvested in Brazil between July 2012 and July 2013, and it was observed that the October harvest presented higher antioxidant and antimicrobial activity, as well as an increase of 1.36 times in thymol and 1.85 times in *p*-cymene content, the major phytochemicals [16]. Additionally, using essential oils in *T. vulgaris*’ and *T. hyemalis*, Jordán et al. (2006) have addressed the effect of the vegetative cycle on the phytochemical profile [35], while Pirbalouti et al. (2013) evaluated wild and cultivated samples of *T. daenensis* essential oil to ascertain the adaptability to crops, where this species produced higher contents of carvacrol or thymol under wild or cultivated growth, respectively [36]. As stated above, the effect of edaphoclimatic parameters on thyme extracts’ phytochemical composition is poorly described. In the present research, we provided new data on the composition variation of aqueous and hydroethanolic extracts of *Thymus carnosus* over a three-year period, comparing both wild plants (harvested at location 1: L1) and plants cultivated in a botanical garden (harvested at location 2: L2), being the geographical locations shown in Figure 1A. L1 corresponds to plants grown in natural conditions, in sand dunes near to the coastline (229 m), and with low elevation (19 m), while L2 corresponds to plants originating in Arrábida National Park (L1) that were cultivated at the botanical garden of the University of Trás-os-Montes and Alto Douro, at an altitude of 451 m and 79 km from the coastline, which adapted to the northern inland climate and soil over a 12-year period. Regarding climate parameters, L2 presents the highest temperature variation, registering lower average temperatures in the winter months and higher average temperatures in summer months when compared to L1, but it overall presents a lower annual average temperature for the 2018–2020 period, as seen in Figure 1B,C. When considering the accumulated precipitation, L2 registered a significantly higher value than L1 (Figure 1B,C).

### 3.1. Extraction Yield, Total Phenolic, Total Flavonoid, and Ortho-Diphenols Content

The extraction yields and results concerning total phenolic (TPC), total flavonoid (TFC), and *ortho*-diphenols (ODC) content, assessed for all extracts of *T. carnosus* harvested in L1 and L2, in the 2018–2020 period, are presented in Table 1. The extraction yield values ranged from 17.82% to 25.43%, being both the lowest and highest yields obtained for hydroethanolic extracts. With the exception of 2020’s harvest at L1, all HE extracts present higher yield compared to the respective AD extracts, as reported for other thyme extracts using the same extraction methods [23,31]. The geographical location also affected the variation of extraction yields, with extracts from L2 presenting higher mean yield values than L1, this difference being more notorious in 2018’s harvest. Concerning inter-year variance, the major differences were observed in 2020, where both HE extracts and AD extracts from L2 presented a decrease in the extraction yield. 

*T. carnosus* AD extracts present higher TPC than the respective HE extract (Table 1), unlike the described for other *Thymus* extracts obtained using the same extraction methodologies (e.g., *T. pulegioides* [24] or *T. mastichina* [23]), in which TPC, assessed by the Folin-Ciocalteau method, was higher in HE extracts. The exception is L2 harvest of 2018, in which no significant differences were found between AD and HE extracts, identical to that reported for *T. fragrantissimus* [30]. However, Folin-Ciocalteau method limitations for TPC quantification are well described, since the reagent can be reduced by other chemical components in the extracts, such as, for example, proteins, thiols, carbohydrates, and amino-acids, where it is likely that AD extracts contain other reducing compounds, which might contribute to TPC overestimation [37]. This observation is supported by ODC and TFC quantification (Table 1), where most HE extracts have significantly higher ODC and TFC contents compared to the respective AD extract, L1 2019′s harvest being an exception. In fact, AD-L1–2019 presented higher TPC, ODC, and TFC than the remaining AD extracts, the ODP and TFC contents being in line with the respective HE extracts, and this extract was highlighted, even having the second lowest extraction yield within AD extracts (Table 1). Regarding the harvest location, overall L1′s extracts present the highest TPC, ODC, and TFC when compared to L2. Inter-year effect proved to induce variations in the extracts’ phytochemical composition. As an example, ODC and TFC inter-year variation can be considered for L1-AD extract, as it is seen that from the first (2018) to the second (2019) year, where the content increased (in mg/g dry plant) 1.40 and 1.34 times, and then it reduced 0.94 and 0.86 times from 2019 to 2020, for ODC and TFC, respectively. In an opposing trend, L2′s HE extract presented the highest ODC and TFC in 2018, being followed by a reduction in the following years, which then present similar values. 

Thus, considering these results, geographical location induces a clearer pattern in the presented data, while climate variations present less predictable results. Regarding HE extracts, a previous report presented TPC values of 41.89 and 45.47 mg/g dry plant for flowering (July) and post-flowering (October) phases, respectively, both higher than the values here presented for *T. carnosus* HE extracts (Table 1). 

### 3.2. Profiling and Quantification of Individual Compounds by HPLC-DAD and HPLC-ESI-MS^n^

**Figure 2:**
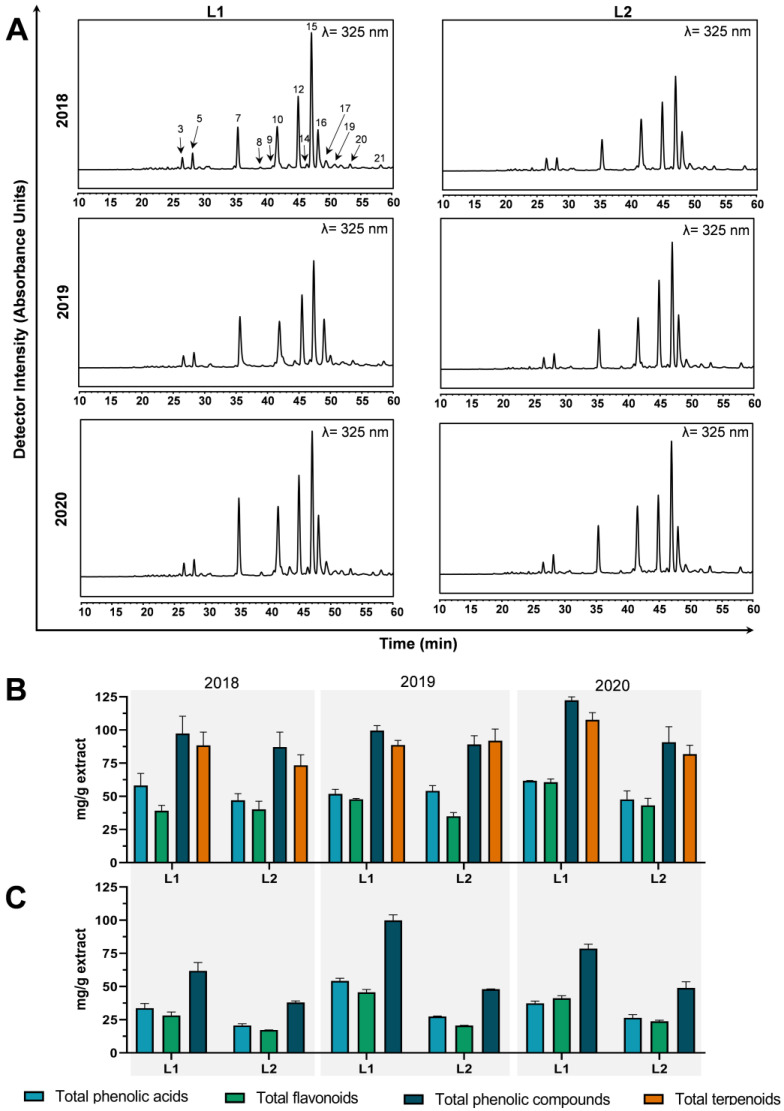
Annual variation (2018–2020 period) of *T. carnosus* phytochemical composition. Chromatograms of phenolic compounds obtained for hydroethanolic extracts (**A**), and, for peak identification and quantification, please refer to Tables S1 and S2. Additionally, there exist variations of total phenolic compounds, phenolic acids, flavonoids, and terpenoids in hydroethanolic (**B**) and aqueous decoction (**C**) extracts obtained by HPLC-DAD analysis. In panel (**A**): 3: caffeic acid; 5: unknown; 7: quercetin-*O*-hexoside; 8: luteolin-*O*-hexoside isomer 1; 9: Luteolin-*O*-hexoside-*O*-pentoside; 10: luteolin-*O*-hexoside isomer 2; 12: salvianolic acid A isomer; 14: acetyl-luteolin-*O*-hexoside-pentoside; 15: rosmarinic acid; 16: salvianolic acid K; 17: salvianolic acid B/E isomer; 19: luteolin-*O*-hexoside-hexoside isomer 1; 20: quercetin-*O*-hexoside-hexuronide; 21: luteolin-*O*-hexoside-hexoside isomer 2.

To better understand the variations in the phytochemical composition, HPLC-DAD-MS^n^ analysis was performed to analyze the variation of the main components of extracts, and that will be relevant to correlate with bioactivities. In Figure 2, HPLC-DAD quantification of total phenolic acids, total flavonoids, total phenols, and total terpenoids, for HE (Figure 2B) and AD (Figure 2C) extracts, was obtained from chromatograms, such as those presented in Figure 2A. The identification of individual compounds was performed with HPLC-ESI-MS^n^ and by comparison of the literature. When considering the sum of all identified and quantified compounds by HPLC-DAD, L1-2020-HE arises as the harvest with the higher content in both phenolics and terpenoids (Figure 2B), while, in AD extracts (Figure 2C), L1-2019-AD is the harvest, presenting higher content in phenolic compounds, as it was also observed in Table 1 for TPC, ODP, and TFC contents in AD extracts. Martins-Gomes, et al. 2018 [9] reported the total phenolics contents assessed by HPLC-DAD, as well as for extracts of *T. carnosus* harvested in July and October 2015, at flowering and post-flowering stages, respectively, with values ranging from 42.92–60.18 mg/g dry AD extract and 146.09–166.45 mg/g dry HE extract. In Table 2, values range from 37.96–99.86 mg/g dry AD extracts, thus most extracts are within the range of those previously reported. However, L1-2019-AD harvest presents a phenolic content above the average, as observed through HPLC-DAD quantification (Figure 2C and Supplementary Table S2). Regarding HE extracts, in the present research, we report phenolic contents within 87.17–122.36 mg/g dry HE extract (Figure 2B and Supplementary Table S1), and all values are lower than the ones previously reported by Martins-Gomes, et al. 2018 [9] for the 2015 harvest. Given the extractability limit of aqueous extraction method, we observe that the contents here presented for AD extracts are in-line with the previous reported [9], while the increased extractability of exhaustive hydroethanolic extraction allows the extraction of all phenolic compounds, revealing that, when compared to July and October 2015 harvests, a harvest in November presents a decrease in total extractable phenolic compounds, most likely arising from changes in the plants’ vegetative phases. 

**Figure 3:**
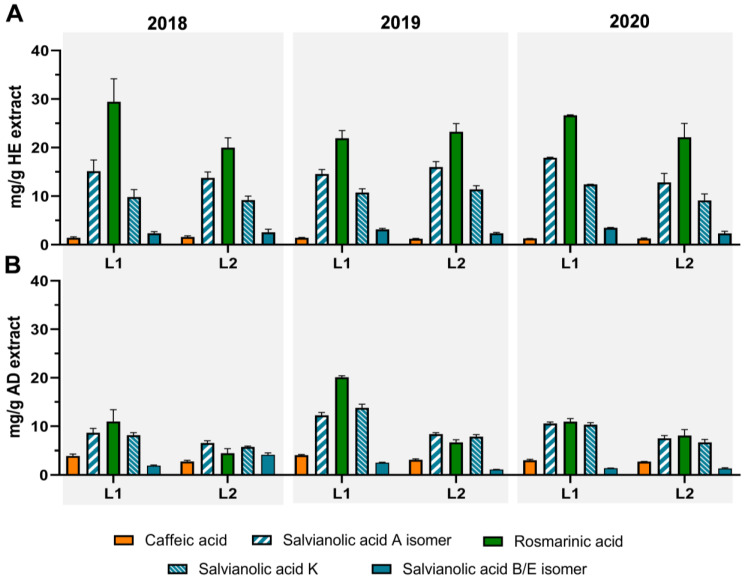
Annual variation (2018–2020 period) of individual phenolic acids in *T. carnosus* hydroethanolic (**A**) and aqueous decoction (**B**) extracts, as obtained by HPLC-DAD analysis.

**Figure 4:**
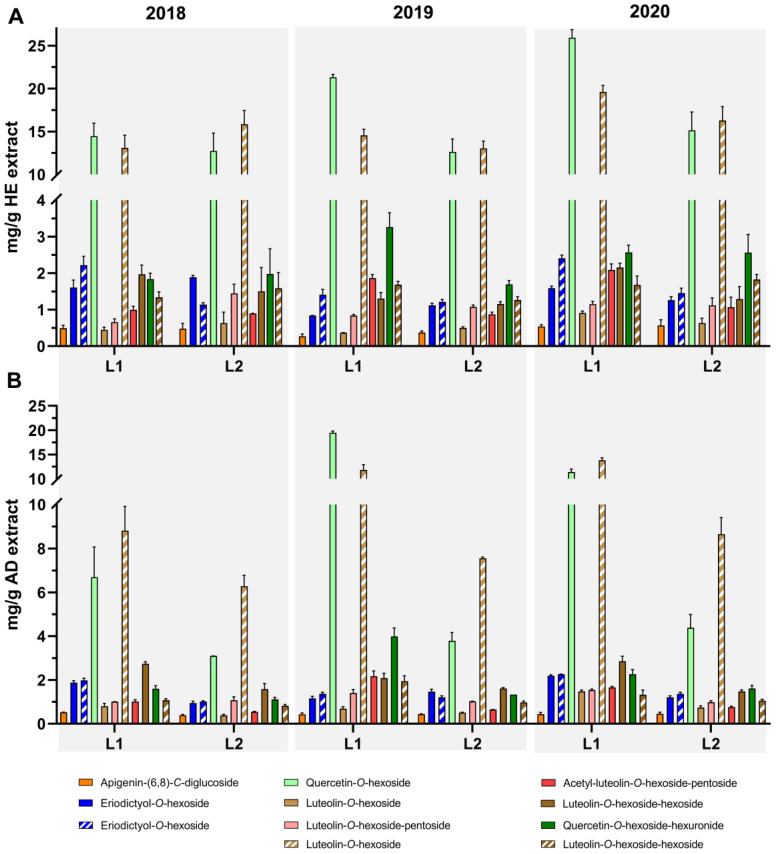
Annual variation (2018–2020 period) of individual flavonoids in *T. carnosus* hydroethanolic (**A**) and aqueous decoction (**B**) extracts obtained by HPLC-DAD analysis.

**Figure 5:**
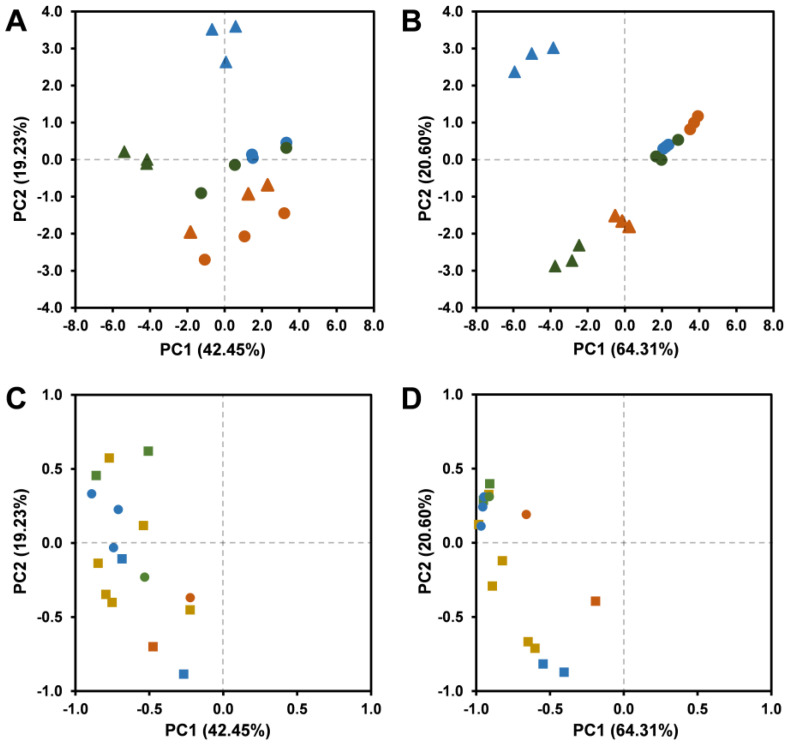
Correlation of *T. carnosus* hydroethanolic (**A**,**C**) and aqueous decoction (**B**,**D**) extracts’ phenolic profile and correlation to phenolic compounds quantified by HPLC-DAD represented as principal component analysis (PCA) scores. Samples were organized using the following shape–colour pattern: (**A**,**B**): L1-▲, L2-●, 2018—orange, 2019—blue, 2020—green; (**C**,**D**): caffeic acid—●, rosmarinic acid—●, salvianolic acids—● apigenin-(6,8)-*C*-diglucoside—■, eriodictyol derivatives—■, quercetin derivatives—■, luteolin derivatives—■.

In Figure 3 (phenolic acids), Figure 4 (flavonoids), Figure 5 (terpenoids), and in Supplementary Tables S1 and S2, we present the quantification of individual compounds identified in *T. carnosus* extracts from L1 and L2 harvests. Overall, *T. carnosus* extracts present a similar profile to extracts reported previously [9] regarding phenolic acids with the presence of RA and salvianolic acids as major components, the identification of two salvianolic acid B/E isomers being identified, and *T. mastichina* [23] and *T. zygis* [29] extracts were also identified, but they were not previously described in *T. carnosus* extracts.

Regarding the effect of the location, edaphoclimatic factors may play a significant role. As described above, L1 is the natural habitat of *T. carnosus*. In fact, extracts obtained from harvests in L1 generally present higher content in phytochemicals. In L2, the average temperature in October and November is lower when compared to L1, which could induce an earlier end of the vegetative phase, thus reducing phytochemicals’ production. A second hypothesis is the effect of drought stress, which is linked to increased secondary metabolite production [38]. Within Portugal’s various edaphoclimatic zones, L1 is within an upper thermomediterranean and dry sub-humid zone, with higher aridity and less precipitation, while L2 is within lower supramediterranean and humid/upper mesomediterranean and humid zones [39]. Thus, the higher availability of water in plants adapted to L2′s climate may justify, in part, the decrease in phytochemicals’ production. 

In a previous report, *T. carnosus* extracts’ phytochemical composition, during flowering and post-flowering stages (July and October 2015, respectively), presented salvianolic acids’ A isomer (SAA isomer) and K (SAK), determined as the major phenolic compounds of both AD and HE extract, ranging from 14.87–27.50 mg/g and 61.92–67.34 mg/g of SAA isomer, as well as 12.53–19.66 mg/g and 38.51–65.33 mg/g of SAK, in AD and HE extracts, respectively [9]. The values for inter-year variance of phenolic acids (Figure 3), for harvests in November, report SAA isomer contents ranging from 6.57–12.24 mg/g in AD extract and 12.83–17.90 mg/g in HE extracts, both lower than the ones from October 2015 harvest [9]. 

Regarding RA content, *T. carnosus* AD extracts content in RA ranges between 4.48 and 20.12 mg/g extract, while HE extracts range between 19.99 and 29.94 mg/g, being the major phenolic acid in these extracts (Figure 3). Considering *T. carnosus* extracts from flowering and post-flowering stage, RA was less predominant when compared to salvianolic acids, with a content of 0.16–4.40 mg/g AD extract and 27.84–29.07 mg/g HE extract [9]. We hypothesize that the much higher amount of salvianolic acids in the plants harvested in July and October 2015 limited RA extraction in AD extracts, whilst, in the present research, a lower content in other phenolic acids allowed a higher RA content in AD extracts, since the overall maximum extractable content (evaluated through HE extracts) is lower or similar to the previous report [9]. Regarding inter-year variance, RA content in HE extracts is overall stable in the three-year period in both locations. On the other hand, AD extracts present an increasing pattern for L2, where RA content increases 1.8 times between 2018 and 2020, whilst, in L1, there were no significant differences between these two years, but L1-2019 presented an increase of 1.83 times in RA content, being much richer in this phenolic acid, as was also observed for salvianolic acids. Raudone, et al. 2017 [21] studied the variation of rosmarinic acid through the various vegetative stages of 8 thyme species (70% hydroethanolic extracts). In all species, the content in RA was decreased in the end of vegetative phase, being, for example, *Thymus praecox* ssp. *arcticus* RA’s content 6.95 times higher in May/June, when compared to August/September [21]. Thus, *T. carnosus* RA content at L2 (19.99–23.24 mg/g extract) increased in plants harvested in November, although it decreased when compared to July (29.07 mg/g extract [9]; between 1.25 and 1.45 times higher), and it was is less expressive when compared to the species described by Raudone, et al. 2017 [21]*,* harvested in September, revealing the stability of RA content in *T. carnosus* extracts even at later harvests.

Nevertheless, the major difference between the data here presented and the previous report is concerning the flavonoids content, presented in Figure 4 and Supplementary Tables S1 and S2. The extracts from L1 and L2 (2018–2020 harvests) exhibited a flavonoid content ranging from 17.24 to 45.69 mg/g for AD extracts and 34.94 to 60.63 mg/g for HE extracts, higher values than the ones reported by Martins-Gomes, et al. 2018 [9] for plants harvested in July and October 2015 (0.92–5.76 mg/g AD extracts and 8.57–9.02 mg/g HE extracts). Contributing to this higher content in flavonoids, the presence of two apigenin derivatives, two eriodictyol derivatives, two quercetin derivatives, and six luteolin derivatives, from which quercetin*-O-*hexoside and luteolin*-O-*hexoside are highlighted as major compounds within the extracts (Figure 4), can be highlighted. Concerning HE extracts, quercetin*-O-*hexoside presented a larger variation in its content in L1. While 2018 harvest had a similar content to L2 harvests in the three years, for the 2020 harvest at L1, we report the highest value for *T. carnosus* extracts with 25.92 mg/g extract.

In accordance with the compounds described above, AD extracts obtained from 2019 harvest at L1 presented not only the highest concentration of quercetin*-O-*hexoside, but also of luteolin*-O-*hexoside, this being last the major phenolic compound of the 2020 harvest at both L1 and L2. As expected, the highest concentrations were obtained for HE extracts, L1-2020-HE once again being highlighted as the extract with higher content in luteolin*-O-*hexoside (19.63 mg/g), and overall, luteolin*-O-*hexoside content in HE extracts ranged between 13.04 and 19.63 mg/g. The concentration of this compound in other *Thymus* species HE extracts was reported for *T. carnosus* [9] (4.61 mg/g extract), *T. pulegioides* [24] (6.31 mg/g extract), and *T. zygis* [29] (3.64 mg/g extract), which were lower than the concentration presented in this research, while being in line with that reported for *T. mastichina* [23] (20.85 mg/g extract), which also presented similar values for quercetin*-O-*hexoside (20.34 mg/g extract) to those here reported for *T. carnosus* HE extracts.

Results presented in Figure 3 and Figure 4 and Supplementary Tables S1 and S2 were used to perform a statistical analysis of inter-year variance based on the quantification of its phenolic individual components through principal component analysis (PCA), which is presented in Figure 5. In Figure 5A,B we present the scatter plots for each extract (and its replicates) as a function of two PCs, explaining 42.45% (PC1) and 19.23% (PC2) of the variation for HE extracts, as well as 64.31% (PC1) and 20.60% (PC2) for AD extracts. PC2 correlates positively with quercetin derivatives in both HE and AD extracts. Figure 5A represents the variance of HE extracts, in which it is possible to observe a major cluster, with clear separation of L1-2019 and L1-2020-HE. This arises from the fact that both extracts present higher content in quercetin*-O-*hexoside, but L1-2019-HE does not present the same pattern for the RA and SAA isomers, which have less correlation with PC1. The remaining HE extracts are mainly grouped within a major cluster, where L2-2018 presents the major correlation to PC1 and lowest correlation to PC2, thus representing a strong correlation with apigenin-(6,8)-*C*-diglucoside and with one eriodictyol derivative and caffeic acid.

Regarding AD extracts (Figure 5B,D), a larger dispersion is observed between harvests, with a cluster being formed by L2 harvests in the 2018–2020 period, and then a strong dispersion is observed within L1 in the three-year harvest. L1-2019-AD clearly stands out as varying according to PC2, and it is correlated negatively with PC1 as a result of its higher RA, salvianolic acids, caffeic acid, and quercetin derivatives content. L1-2020-AD correlates poorly with PC2, being that these extracts are differentiated by a higher content in luteolin derivatives. L1-2018-AD, presents higher content of apigenin-(6,8)-*C*-diglucoside, the second highest caffeic acid concentration, but lower contents of quercetin derivatives, which assume a distinct position from the other L1 harvests. Of all the analyzed extracts, the AD extracts from L2 present the least variation among them.

**Figure 6:**
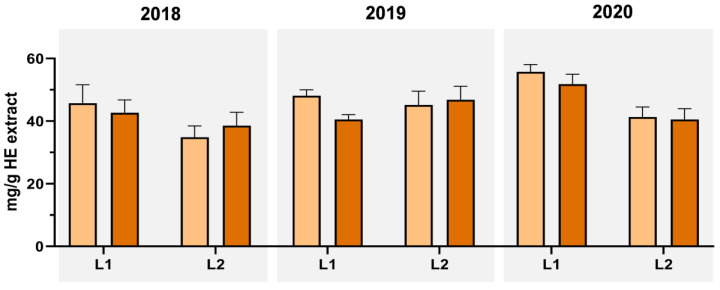
Annual variation (2018–2020 period) of oleanolic (OA) and ursolic (UA) acids in *T. carnosus* hydroethanolic extracts obtained by HPLC-DAD analysis.

Concerning the pentacyclic diterpenoids, (Figure 6 and Supplementary Table S1), we observed that L1 overall presented a higher content of both OA and UA when compared to L2 (with exception of UA content in 2019), and within the 2018–2020 period, 2020′s harvest presented the highest content of terpenoids (OA: 55.80 mg/g and UA: 51.84 mg/g). Oleanolic (OA) and ursolic (UA) acid have been described in *T. carnosus* HE extracts for the first time by Martins-Gomes, et al. 2018 [9], who reported a high content of both pentacyclic triterpenoids for both flowering stage (OA: 39.43 mg/g and UA: 75.17 mg/g) and post-flowering stage (OA: 41.74 mg/g and UA: 75.76 mg/g). A second conclusion can be retrieved when comparing vegetative phases, where, while OA content is similar to those presented by Martins-Gomes, et al. 2018 [9], the harvests of July and October favour a higher content in UA, whose highest content was 1.45 times higher than the observed for L1-2020-HE. Therefore, changes in the vegetative phase may induce differences in the production of such phytochemicals, that, similar to the ones observed for the phenolic acids described above, have a reduced concentration in November. Nevertheless, this comparison arises from an initial study from Martins-Gomes, et al. 2018 [9] with a single year analyzed. Additional studies for inter-year variation are essential to ascertain *T. carnosus* extracts’ phytochemical variation in response to vegetative phase changes and climate adaptation. It is possible to observe that November also benefits the production of flavonoids derivatives, and L1 (the location from where the species is native) presents overall higher content in the phytochemicals here analyzed, most likely a result of a better adaptation to the edaphoclimatic conditions.

The variation of oleanolic and ursolic acid was previously evaluated in the *Thymus* genus, in a single year harvest of plants grown in the same location, to study the variation induced by the vegetative stage in various species. In the various *Thymus* species analyzed by Raudone, et al. 2017 [21], *Thymus sibtorpii* Benth., *Thymus austriacus* Bernh. ex Rchb., *Thymus* × *oblongifolius* Opiz, and *Thymus* × *citriodorus* presented the highest content of OA and UA during the flowering stage, while *Thymus serpyllum* L., *Thymus pulegioides,* and *Thymus longicaulis* presented the highest content at the end of vegetative phase. Thus, we observe that pentacyclic terpenoids variation is also dependent on inter-species variation motivated by genetic variations, in addition to the edaphoclimatic parameters.

### 3.3. In Vitro Antioxidant Activity Assessment

The variation of herbal extracts’ antioxidant capacity induced by biotic and abiotic factors has been previously addressed for several species, as the antioxidant activity is one of the main sought-after bioactivities for these products. We report here, for the first time, the study of *T. carnosus* extracts antioxidant activity variation as a result of different edaphoclimatic parameters and inter-year variance. The results for ABTS^•+^, ^•^OH, NO^•^, O_2_^•−^, and β-carotene bleaching assays are presented in Table 2.

ABTS^•+^ scavenging was used as a reference antioxidant assay to compare both the extracts here reported, but also to allow the comparison with other species. In Table 2, it is possible to observe that ABTS^•+^ scavenging results ranged between 0.15 and 0.25 mmol Trolox equivalent/DP. The highest scavenging value was obtained for L1-AD-2019, which also presented the highest TPC content (Table 1), and thus it correlated with the ABTS^•+^ scavenging for a higher content in reducing compounds, including phenolics and polysaccharides, for example. The lowest value was obtained for L2-HE-2020. Martins-Gomes, et al. 2018 [9], for *T. carnosus,* harvested in July October 2015, reported inhibitions of 0.14 and 0.21 mmol Trolox eq./DP, for AD and HE extracts, respectively, thus being comparable to the results here obtained. When compared to other *Thymus* species, our results align with the values reported for HE extracts *T. vulgaris* (0.22 mmol Trolox eq./DP) [31] and *T. mastichina* (0.20 mmol Trolox eq./DP) [23], whilst *T. zygis* (0.25 mmol Trolox eq./DP) presented higher inhibition for HE extracts. Concerning AD extracts, the *T. carnosus* extracts here presented performed better than *T.* x *citriodorus* (0.11 mmol Trolox eq./DP) [31] and *T. mastichina* (0.081 mmol Trolox eq./DP) [23], species commonly used for human consumption in herbal teas and as condiments. Concerning the inter-year variation, in AD extracts, L1-2018 presented an ABTS^•+^ scavenging activity significantly lower than the respective harvests in 2019 and 2020, whilst no significant variations were observed for L2. For the HE extracts, L1′s harvests did not present significant variations between years, however, in L2′s harvests, the 2018-HE-L2 extract presented significantly higher ABTS^•+^ scavenging. When comparing the variation induced by the different locations, AD extracts scavenging was only statistically different in 2020, where L1-2020-AD presented higher antioxidant activity. In the HE extracts, significant differences were observed in 2018, as L2-2018-HE presented higher scavenging.

**Figure 7:**
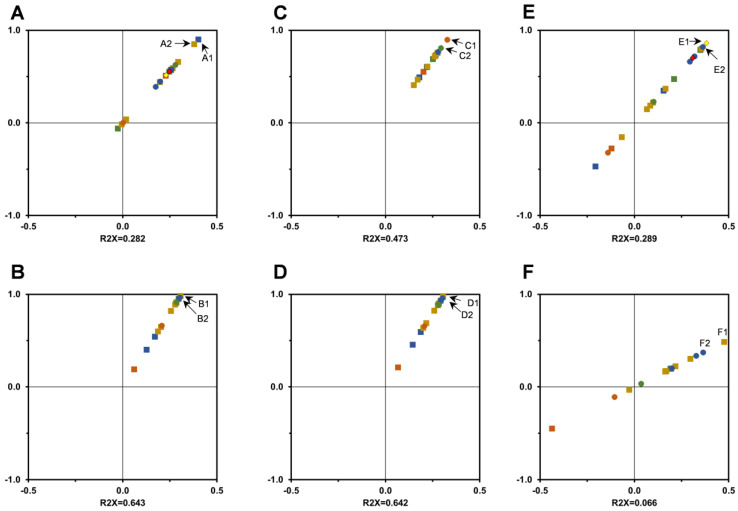
Correlation between individual phytochemicals and ABTS^•+^ scavenging ((**A**): HE extract; (**B**): AD extract), hydroxyl radical scavenging ((**C**): AD extract), nitric oxide scavenging ((**D**): AD extract) and β-carotene bleaching inhibition ((**E**): HE extract; (**F**): AD extract), by *T. carnosus* extracts presented as score-plots obtained by OPLS-DA model analysis. Caffeic acid—●, rosmarinic acid—●, salvianolic acids—● apigenin-(6,8)-*C*-diglucoside—■, eriodictyol derivatives—■, quercetin derivatives—■, luteolin derivatives—■, oleanolic acid—♦; ursolic acid—♦. A1: eriodictyol-*O*-hexoside isomer 2; A2: luteolin-*O*-hexoside-hexoside isomer 1; B1/D1: acetyl-luteolin-*O*-hexoside-pentoside; B2/D2/F2: salvianolic acid A isomer; C1: caffeic acid; C2: rosmarinic acid; E1: oleanolic acid; E2: salvianolic acid K; F1: luteolin-*O*-hexoside-pentoside. Model validation is presented in supplementary material (Figures S2–S4).

Aiming to study the correlation between the various individual phytochemicals and each bioactivity, score-plots similar to those presented in Figure 5 for PCA were obtained using the OPLS-DA (orthogonal partial least squares discriminant analysis) model. This analysis allows an easier interpretation and presents advantages to data sets comprising a higher number of observations than the number of variables, being proposed as a promising tool to highlight statistically significant components in comparable data sets [28,40]. The OPLS-DA model uses an orthogonal signal correction filter to identify the variations related to the prediction of a quantitative response from the variations not related to the prediction [40]. We present the correlations obtained by the OPLS-DA model when significant correlations were observed, and the models present an adequate degree of validation (models’ validation is presented in the Supplementary material). Using data obtained in ABTS^•+^ scavenging assays, score-plots were obtained using the OPLS-DA model, which are presented in Figure 7. Most of phytochemicals identified and quantified in *T. carnosus* extracts positively correlate with the ABTS^•+^ scavenging (Figure 7A). Quercetin-*O*-hexoside-hexuronide, luteolin-*O*-hexoside-pentoside, and caffeic acid present the lowest correlation with this antioxidant activity in HE extracts. CA content does not present a large variation influenced by either year or location. Quercetin-*O*-hexoside-hexuronide is present at the highest concentration in L1-2019-HE, L1-2020-HE, and L2-2020-HE, which are within the extracts that present lowest ABTS^•+^ scavenging, thus being the components that least explain the increase in scavenging as provided by OPLS-DA model. Eriodictyol-*O*-hexoside isomer 2 and luteolin-*O*-hexoside-hexoside isomer 1 are the components which better explain the variation observed. Regarding AD extracts (Figure 7B), acetyl-luteolin-*O*-hexoside-pentoside, SAK, and SAA iso were the phytochemicals with highest contribution to ABTS^•+^ scavenging variance. L1-2019-AD and L1-2020-AD present the highest content of these three phytochemicals among the AD extracts studied, thus supporting the correlation.

In addition to ABTS^•+^, the scavenging of biologically relevant radicals was also performed, namely, for radicals such as ^•^OH, NO^•^, and O_2_^•−^, as well as β-carotene bleaching as a model for lipid peroxidation. The capacity of extracts in ^•^OH scavenging in the absence of EDTA was dependent on their geographical origin, as L1 presented higher activity, obtaining inhibitions greater than 50%, and L1-AD-2019 produced the lowest IC_50_ (0.8 mg/mL; Table 2), once again correlating to the higher content in phenolic compounds. When analyzing inter-year variance, L1-2020-AD presented a significantly lower inhibition when compared to 2018 and 2019, whilst, in L2, 2019′s harvest presented a higher inhibition, although not significantly higher than L2-2018-AD and L2-2020-AD. In the presence of EDTA, all extracts performed in a similar manner, with approximately 30% of inhibition as the maximum value obtained in these conditions. In similar manner to that described for ABTS, the OPLS-DA model was applied to data obtained from ^•^OH scavenging assay in the absence of EDTA and is presented in Figure 7C. All phytochemicals correlate positively with ^•^OH scavenging, with phenolic acids (by this order: CA, RA, SAA iso and SAK) being the components with the highest influence in ^•^OH scavenging, in line with the phytochemical composition obtained by HPLC-DAD, where overall extracts from L1 present higher content in these phenolic acids. 

The capacity of extracts in NO^•^ scavenging followed a pattern identical to ^•^OH scavenging, as presented in Table 2. L1-AD-2019 produced both the highest inhibition (73.31%) and the lowest IC_50_ (0.57 mg/mL) values. Similarly, L1 extracts presented higher ability to scavenge this radical, most likely due to higher content of phenolic compounds. When compared to a previous report of NO^•^ scavenging by *T. carnosus* extracts [9] (41.79%; 1 mg/mL), our extracts presented higher potential, all achieving IC_50_ values bellow 1 mg/mL. Considering the different harvests in the same location, extracts from plants harvested in 2019 presented higher inhibition, at 1 mg/mL, as well as lower IC_50_ values (Table 2). OPLS-DA analysis for NO^•^ scavenging (Figure 7D) also revealed a similar pattern to ABTS^•+^ scavenging, with all compounds correlating with the activity, supported by the higher content in phytochemicals observed in L1-AD-2019, producing the highest inhibition and lowest IC_50_. The compound with higher correlation is also identical to ABTS assay, acetyl-luteolin-*O*-hexoside-pentoside, whose highest content is observed in L1-2019-AD. 

In this research, we report, for the first time, *T. carnosus* extracts’ ability to scavenge O_2_^•−^, as well as the potential to reduce lipid peroxidation (Table 2). All extracts displayed significant capacity to scavenge O_2_^•−^ using the xanthine oxidase/NBT assay. Overall, AD extracts present higher inhibition mean values, but these are only significant in the L1-2019 harvest. The inhibition values ranged between 31.41% (L1-HE-2018) and 49.72% (L1-AD-2019) for the most efficient extract (Table 2). Unlike for ^•^OH or NO^•^ radicals’ scavenging, there is no pattern for the location effect concerning the extracts’ scavenging of O_2_^•−^, since, when comparing locations, L1-2018 performed more poorly than L2-2018, and, when comparing inter-year effect, it presented less activity than L1-2019 and L1-2020. L2-2018 presented less O_2_^•−^ scavenging than the respective harvest at L1 and the other L2 harvests. *T. fragrantissimus* extracts capacity to scavenge this radical indicated an inhibition of 48.81% for AD extracts, as well as 49.63% for HE extracts [30], values similar to those obtained in the 2019 harvest (Table 2), which are also in line with results obtained for *T. zygis* methanolic extracts (40.3%) [41] and for *T. vulgaris* aqueous extracts (~45%) [42].

Concerning lipid peroxidation, as seen in Table 2, all extracts were able to greatly inhibit β-carotene bleaching, with the most effective extract being L1-HE-2020, as well as being the extract with higher phenolic and terpenoid compounds quantified by HPLC-DAD, with the maximum inhibition of 96.25% at 1 mg/mL. Overall, HE extracts presented higher maximum inhibitions, with the exception of L2-2018, but the IC_50_ revealed a different pattern. Only in L1-2019 and L1-2020 were significant differences observed concerning the IC_50_ values, which were found between extraction methods, with AD extracts presenting lower IC_50_ values. Thus, HE extracts, most likely due to a higher phenolic content, generally achieve higher inhibition, whilst AD extracts achieve about 50% of inhibition with equal or slightly lower concentrations (Table 2). Concerning inter-year variation, in both AD and HE extracts, an increase in β-carotene bleaching inhibition is observed with the year of harvest, as 2018 < 2019 < 2020, whilst, in L2, the harvests of 2019 present higher inhibition. OPLS-DA model analysis (Figure 7E) revealed that, in HE extracts, compounds with increased hydrophobicity among the components of the extracts, namely, oleanolic acid, ursolic acid, SAK, and acetyl-luteolin-*O*-hexoside-pentoside, positively influence the extracts’ ability to inhibit the bleaching. In AD extracts, with a lesser content in these phytochemicals, luteolin-*O*-hexoside-pentoside and SAA isomer present the highest contributions to explain the variance observed.

Concerning the β-carotene bleaching assay, *T. mastichina* extracts have been reported to produce an IC_50_ of 0.9 mg/mL [43], a much higher value than the ones reported in the present study (Table 2). Nevertheless, extracts of *T. pulegioides* [44], *T. caespititus* [25], and *T. pseudolanuginosus* [25] presents IC_50_ values of 30 µg/mL, 6.1 µg/mL, and 2.4 µg/mL, respectively, thus showing higher potential to reduce lipid peroxidation when compared to *T. carnosus* extracts.

In sum, both extraction methods, year of harvest and location, induce significant variations in the overall antioxidant capacity of *T. carnosus* extracts. In addition, this activity, although reported for the various tested radicals, reveals that the differences in each extract’s composition (within each extraction method) induce variations in the scavenging ability, highlighting a capacity that is dependent of the radical selected. Considering its health-promoting effects, it is here demonstrated that *T. carnosus* bioactive components might be effective tolls to counter the oxidative stress activity in biological systems, which still requires further studies.

Various extracts of *Gingko biloba* obtained from material harvested in various locations in India over three different seasons showed that, in addition to a direct correlation between the phenolic content and antioxidant activity, the highest values of both parameters were reported in the autumn, where the antioxidant activity was expressed as ABTS and DPPH radicals scavenging [45]. In *Thymus* spp., variation of antioxidant activity induced by seasonal changes and developmental stages was also reported. Using essential oils from *T. vulgaris* harvested in Brazil over a twelve months period, Lemos*, et al.* 2017 [16] observed that ABTS and DPPH radicals scavenging was higher in Spring, correlating with a high content of thymol and carvacrol. Regarding the developmental stages, aqueous extracts from *Thymus hirtus* Boiss. and Reut., harvested in Tunisia, in a single year, in various vegetative phases and rich in catechin and epicatechin, were reported to present the highest antioxidant activity at flowering stage [46]. In *T. longicaulis* methanolic extracts, rich in RA, salvianolic acid K and luteolin derivatives, having a more similar phytochemical profile to the extracts of *T. carnosus* here reported, it was observed that, in a nine months period, the scavenging activity of ABTS and DPPH radical varied greatly, with IC_50_ values ranging 8.91–72.01 µg/mL and 9.50–64.61 µg/mL for ABTS and DPPH radicals, respectively, which provide a new insight into a significant variation of the antioxidant activity dependent of the date of harvest [20]. Nevertheless, even extracts from commonly consumed species, such as *T. vulgaris,* present reduced information regarding the variation of bioactivities induced by changes in the harvests’ location and date.

### 3.4. Enzymatic Inhibition Assays

In Table 3 are presented the results for *T. carnosus* extracts’ inhibition of key enzymes often described as druggable targets aiming neuroprotection (AChE and tyrosinase), anti-aging (tyrosinase and elastase), and diabetes management through lowering the intestinal absorption of glucose (α-amylase and α-glucosidase). With the exception of reports on *T. carnosus* essential oil activity in AChE inhibition [47], we present here, for the first time, the evaluation of *T. carnosus* AD and HE extracts’ anti-enzymatic activity described above.

Concerning potential neuroprotection effect, all extracts were effective in inhibiting AChE and tyrosinase activity, the most efficient extracts being L1-2019-AD for AChE and L2-HE-2020 for tyrosinase. All AD extracts presented significantly higher capacity to inhibit AChE when compared to the respective HE extract, despite the lower content in phenolic compounds (quantified by HPLC-DAD; Figure 2, Figure 3 and Figure 4). This may be explained by the presence of other water-soluble non-phenolic components that are extracted by the AD method that present the capacity to inhibit this enzyme. *T. carnosus* essential oil has been reported to inhibit AChE activity, with an IC_50_ of 0.72 mg/mL [47], and thus it is in-line with the extracts here present, whose best inhibition value at 1 mg/mL (L1-2019-AD) was 61.47% inhibition. Rich in RA, *T. pulegioides* extracts presented a higher efficiency in inhibiting AChE, being able to reduce the enzyme activity in almost 90% at 0.5 mg/mL [24]. *T. vulgaris* ethanolic extracts produced an inhibition above 75% at 1 mg/mL [48], which is also higher than the values here reported (Table 3). A similar comparison can be performed for *T. serpyllum* ethanolic and aqueous extracts, which inhibited 50% of AChE activity at 0.25 and 0.35 mg/mL, respectively [49]. On the other hand, *T. fragrantissimus* extracts produced lower inhibitions, being able to inhibit only 27.30% at 0.5 mg/mL [30]. 

**Figure 8:**
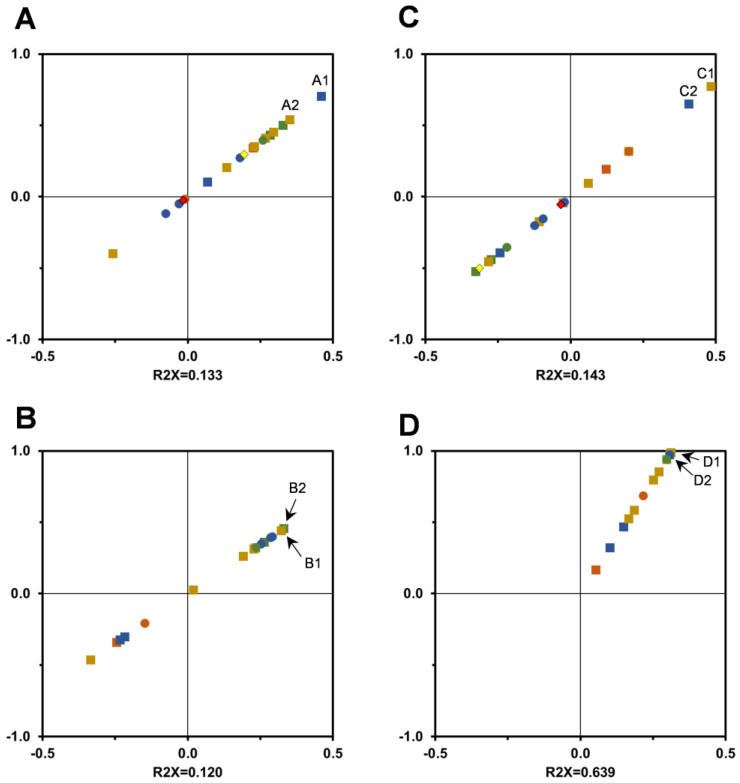
Correlation between individual phytochemicals and Anti-AChE ((**A**): HE extract; (**B**): AD extract) and α-glucosidase ((**C**): HE extract; (**D**): AD extract), by *T. carnosus* extracts presented as score-plots obtained by OPLS-DA model analysis. Caffeic acid—●, rosmarinic acid—●, salvianolic acids—● apigenin-(6,8)-*C*-diglucoside—■, eriodictyol derivatives—■, quercetin derivatives—■, luteolin derivatives—■, oleanolic acid—♦; ursolic acid—♦. A1: eriodictyol-*O*-hexoside isomer 2; A2/B1: luteolin-*O*-hexoside-hexoside isomer 1; B2: quercetin-*O*-hexoside-hexuronide; C1: luteolin-*O*-hexoside-pentoside; C2: eriodictyol-*O*-hexoside isomer 1; D1: acetyl-luteolin-*O*-hexoside-pentoside; D2: salvianolic acid K. Model validation is presented in supplementary material (Figures S5 and S6).

The OPLS-DA model was used to evaluate the influence of individual phytochemicals present in *T. carnosus* extract in AChE inhibition variation, as is presented in Figure 8A for HE extracts and Figure 8B for AD extracts. For HE extracts, eriodictyol-*O*-hexoside isomer 2, luteolin-*O*-hexoside-hexoside isomer 1, acetyl-luteolin-*O*-hexoside-pentoside, and the two quercetin derivatives are the individual components which most contribute to explain the increase in AChE inhibition. Quercetin was used as the positive control for this assay, as it inhibits AChE activity significantly, and thus quercetin’s glycoside derivatives identified in *T. carnosus* HE extracts likely also present inhibitory activity. In AD extracts, luteolin-*O*-hexoside-hexoside isomer 1 is also a major contributor to the variance observed in AChE inhibition, namely, in its increase.

Considering the location effect, L1, AD, and HE extracts, from the 2018 and 2019 harvests, produce greater AChE inhibitions. For the 2020 harvest, identical AChE inhibition was obtained with L1 and L2 HE extracts (*p* > 0.05). However, L2-AD-2020 has a greater capacity to inhibit AChE than L1-AD-2020. Regarding the inter-year variation, L1-HE extracts produced no variation in AChE inhibition, while L1-AD extracts harvested in 2020 produced a significant decrease in AChE inhibition. For L2′s extracts, AD extracts’ anti-AChE inhibition increases in 2019 and 2020, when compared to 2018, and HE extracts also present significantly higher inhibition in 2020. 

The inhibition of tyrosinase activity by *T. carnosus* extracts is dependent on their phytochemical content, since the HE extracts always present higher inhibition than the AD extracts. However, L1-2020-HE, the extract with higher phenolic acids, flavonoids, and terpenoids quantified by HPLC-DAD did not produce the highest inhibitory effect. Overall, the extracts present potential for neuroprotection, with efficacy varying based on location and year of harvest, but without a defined pattern (Table 3). *T. fragrantissimus* AD extracts were also able to inhibit tyrosinase (56.30% at 0.5 mg/mL) [30], while *T. pulegioides* presented a much higher inhibition capacity (93.72% at 0.5 mg/mL) [24]. Both species showed higher potential than *T. carnosus*, whose extracts were able to inhibit a maximum of 42.54% for L2-2020-HE (Table 3).

*T. carnosus* extracts presented a poor efficacy regarding elastase inhibition, thus not being the primary potential choice for anti-aging applications, unlike *T. fragrantissimus* HE extracts that inhibited 48.69% of elastase activity [30]. *T. carnosus* extracts here presented (Table 3) showed a pattern similar to *T. pulegioides* [24], where no elastase inhibition capacity was observed for HE extracts, but AD extracts were able to reduce elastase activity. The highest inhibition obtained in the present research was 7.31% using L1-2019-AD (Table 3), which is also the best extract for AChE inhibition.

As with the results of tyrosinase inhibition, the inhibition of α-amylase activity is dependent on the phenolic and terpenoids content, since the inhibition induced by HE extracts is significantly greater than the respective AD extracts. Nevertheless, once again L1-2020-HE was not the best performing extract, suggesting that the overall composition and ratio between all components may play a role, which are opposed to the simple higher content in each individual component. The extracts were not able to induce a reduction in α-amylase activity greater than 6%, which are results in line with those obtained by Taghouti et al. (2018) using *T. pulegioides* extracts [24]. On the other hand, *T. carnosus’* potential for anti-diabetic applications may be achievable through α-glucosidase inhibition, where a maximum inhibition of 27.37% was achieved for L1-2019-AD. 

Regarding the location, AD extracts from L1 (in all harvests) produced, on average, higher inhibition of tyrosinase activity than AD extracts from L2. While, for HE extracts, significantly higher inhibition was produced by extracts from L2 harvests in 2018 and 2019, but not in 2020. Regarding the harvest year variation, it was observed that AD-2019-L1 extract produced higher tyrosinase inhibition than the other AD extracts, while the best HE extract was from L2 during the harvest of 2020 when compared to the other harvest years. 

The extraction method influenced anti-glucosidase activity, as well as L2-HE extracts from 2018 and 2020 harvests, which show higher content in phytochemicals and also show higher inhibition capacity. In an opposed pattern, L1-2019-AD produced significantly higher α-glucosidade inhibition when compared to the respective HE extract (Table 3). 

The contribute of individual phytochemicals to α-glucosidase inhibition variation was also evaluated by OPLS-DA model, and it presented in Figure 8C for HE extracts and Figure 8D for AD extracts.

As determined by OPLS-DA model (Figure 8), the higher contribute to α-glucosidase inhibition variation produced by HE extracts is due to the presence of luteolin-*O*-hexoside-pentoside, eriodictyol-*O*-hexoside isomer 1, and quercetin-*O*-hexoside-hexuronide, which may be connect to the presence of sugar residues in these glycoside derivatives. Future analysis of the role of individual compounds in this bioactivity should address the effect of the presence of sugar residues in comparison to the aglycone to ascertain if these moieties have higher affinity to the enzyme’s active site. In fact, L2-2018-HE, the HE extract with higher α-glucosidase inhibition, is also the extract with higher content of luteolin-*O*-hexoside-pentoside and of the first eriodictyol-*O*-hexoside isomer identified, which correlates with the OPLS-DA model. For AD extracts, the compounds whose variation better explain the inhibition pattern are acetyl-luteolin-*O*-hexoside-pentoside and the salvianolic acids, factors correlated with the increase in phytochemicals observed in L1-2019-AD, which produced a significantly higher α-glucosidase inhibition. 

At 1 mg/mL, *T. carnosus* extracts presented an α-glucosidase inhibition ~2.16 times and 2.71 times higher than *T. fragrantissimus* [30] and *T. pulegioides* [24] extracts at 0.5 mg/mL (the only concentration tested). Aqueous extracts of *T. vulgaris* (concentration not mentioned) were reported to inhibit 4% and 20% of α-amylase and α-glucosidase, respectively [50], which are also in-line with the results were reported. Therefore, *T. carnosus* extracts present a moderate potential for the reduction of post-prandial blood sugar uptake by inhibiting α-glucosidase. Moreover, despite the variations induced by the unique phytochemical composition of each extract, influenced by harvest year, location, and extraction method, all extracts present neuroprotective and anti-diabetic activity, and thus *T. carnosus* can be a reliable functional food for these applications. Although there are few reports for salvianolic acids’ ability to inhibit these key enzymes, salvianolic acid B presents potential to inhibit AChE, and salvianolic acid C has been shown to inhibit α-glucosidase [51,52]. RA and pentacyclic triterpenoids have been shown to inhibit AChE, α-glucosidase, and α-amylase activity, and thus the harvest at earlier vegetative stages may increase this potential effect [48,53,54,55]. Thus, it is also relevant for future studies to further unveil the potential effects of *T. carnosus* extracts bioactivities, especially at the gastrointestinal tract, since AD extracts present a wide array of potential bioactivities, and this is reported in the present research.

## 4. Conclusions

Considering the high number of species used for its health-promoting bioactivities and the large number of pharmaceutical products obtained from plants, it is essential to increase the screening for new medicinal plants and phytochemicals with pharmaceutical potential. In addition, due to the increasing number of species threatened by climate and anthropogenic factors, there is a risk of losing the potential applications of a high number of species that were never considered for screening. Aiming to increase the interest in *T. carnosus* and encourage a sustainable crop, in the present research we complement the previous work related to *T. carnosus* extracts phytochemical composition and anti-proliferative activity by further analyzing its neuroprotective, anti-aging, and antidiabetic activity, complementing the antioxidant activity and evaluating the effect of edaphoclimatic parameters in the phytochemical composition and bioactivities. In addition to a unique phytochemical composition, *T. carnosus* extracts are a promising source of rosmarinic and salvianolic acids, as well as glycoside derivatives of various flavonoids, where the vegetative stage plays a significant role in the concentration of each phenolics subclass.

Despite the variations in the phytochemical profile, the extracts maintained significant antioxidant and neuroprotective activities and moderate anti-diabetic activity in the three-year period, thus revealing the potential to induce health-promoting activities when included in the diet, as demonstrated by the AD extracts, but also as a source of phytochemicals. Further studies on *T. carnosus* extracts should be performed to complement the information available, such as studies on its antioxidant and anti-diabetic potential in in vitro and in vivo experimental models.

## Figures and Tables

**Table 1:** Extraction yields and total phenolics, *ortho*-diphenols, and flavonoid content of *Thymus carnosus* extracts.

Year	Loc.	E. M.	Extraction Yield	Total Phenols (mg Caffeic Acid eq./g)		*Ortho*-Diphenols (mg Caffeic Acid eq./g)		Total Flavonoids (mg Catechin eq./g)	
			% *w*/*w*	mg/g Extract	mg/g DP	mg/g Extract	mg/g DP	mg/g Extract	mg/g Dry DP
2018	L1	AD	17.90 ± 0.70 ^Aa^	210.93 ± 7.45	37.76 ± 1.33 ^Aa*^	95.43 ± 5.44	17.08 ± 0.97 ^Aa*^	146.85 ± 6.64	26.29 ± 1.19 ^Aa*^
		HE	18.98 ± 0.77 ^ABa^	167.60 ± 14.03	31.81 ± 2.66 ^Aa*^	118.29 ± 6.97	22.45 ± 1.32 ^Aa*^	149.79 ± 0.38	28.43 ± 0.07 ^Aa*^
	L2	AD	22.78 ± 1.77 ^Ab^	164.74 ± 1.84	37.53 ± 0.42 ^ABa^	67.50 ± 1.75	15.38 ± 0.40 ^Ab*^	104.62 ± 8.43	23.83 ± 1.92 ^Aa*^
		HE	25.43 ± 2.83 ^Ab^	152.90 ± 13.89	38.88 ± 3.53 ^Aa^	94.50 ± 9.65	24.03 ± 2.45 ^Aa*^	146.35 ± 3.58	37.22 ± 0.9 ^Ab*^
2019	L1	AD	18.98 ± 1.21 ^Aa^	258.65 ± 9.06	49.09 ± 1.72 ^Ba*^	126.11 ± 1.97	23.93 ± 0.37 ^Ba^	186.35 ± 5.16	35.37 ± 0.98 ^Aa*^
		HE	21.35 ± 1.91 ^Aa^	173.52 ± 14.04	37.05 ± 2.98 ^Aa*^	110.57 ± 15.75	23.61 ± 3.36 ^Aa^	144.93 ± 1.38	30.94 ± 0.29 ^Ba*^
	L2	AD	21.03 ± 0.52 ^Aa^	194.90 ± 16.08	40.99 ± 3.38 ^Ab*^	82.50 ± 5.25	17.35 ± 1.10 ^Ab*^	131.92 ± 6.26	27.74 ± 1.32 ^Bb^
		HE	22.24 ± 1.53 ^ABa^	150.42 ± 17.31	33.45 ± 3.85 ^ABb*^	93.00 ± 7.27	20.68 ± 1.62 ^ABa*^	123.70 ± 8.44	27.51 ± 1.88 ^Bb^
2020	L1	AD	19.08 ± 0.54 ^Aa^	242.81 ± 5.95	46.33 ± 1.13 ^Ba*^	118.43 ± 3.54	22.60 ± 0.67 ^Ba^	162.38 ± 4.38	30.98 ± 0.84 ^Ca^
		HE	17.82 ± 0.68 ^Ba^	202.34 ± 7.16	36.06 ± 1.28 ^Aa*^	127.07 ± 6.54	22.64 ± 1.17 ^Aa^	176.92 ± 1.84	31.53 ± 0.34 ^Ba^
	L2	AD	19.27 ± 0.13 ^Ba^	184.59 ± 2.65	35.57 ± 0.51 ^Bb*^	80.36 ± 2.61	15.49 ± 0.50 ^Ab*^	118.08 ± 5.51	22.75 ± 1.06 ^Ab*^
		HE	19.61 ± 2.03 ^Ba^	154.62 ± 6.45	30.32 ± 1.27 ^Bb*^	97.43 ± 1.61	19.11 ± 0.31 ^Bb*^	135.77 ± 5.89	26.62 ± 1.15 ^Bb*^

Abbreviations: AD: aqueous decoction and HE: hydroethanolic extractions; Loc.: location; E.M.: extraction method. Tukey’s post hoc test was performed to analyze significant statistical differences between extraction methods (E. M.) for the same year and location (*) between years for the same location and extraction method (different capital letters) and between locations for the same year and extraction methods (different lowercase letters) if *p* < 0.05, in mg/g dry plant. Results are presented as mean ± standard deviation (n = 3).

**Table 2:** Evaluation of *T. carnosus* extracts antioxidant scavenging against ABTS, hydroxyl, nitric oxide radicals, and β-carotene bleaching assay.

Year	Loc.	E.M.	ABTS^•+^	Hydroxyl (^•^OH)				Nitric Oxide (NO^•^)		Superoxide (O_2_^•−^)		β-Carotene Bleaching	
			mmol Trolox eq/DP	% Inhibition wo/EDTA	IC_50_ (mg/mL)	% Inhibition w/EDTA	IC_50_	% Inhibition	IC_50_ (mg/mL)	% Inhibition	IC_50_ (mg/mL)	% Inhibition	IC_50_ (mg/mL)
2018	L1	AD	0.19 ± 0.01 ^Aa^*	54.92 ± 2.86 ^ABa^	0.87 ± 0.04 ^AB^	33.77 ± 0.78 ^Aa^	-	65.89 ± 1.01 ^Aa^	0.74 ± 0.01 ^Aa^	33.83 ± 4.14 ^Aa^	1.67 ± 0.16 ^Aa^	70.33 ± 3.46 ^Aa^	0.15 ± 0.02 ^ABa^
		HE	0.16 ± 0.01 ^Aa^*	-	-	-	-	-	-	31.41 ± 5.60 ^Aa^	1.67 ± 0.19 ^Aa^	77.95 ± 8.82 ^Aa^	0.16 ± 0.02 ^Aa^
	L2	AD	0.20 ± 0.01 ^Aa^	38.51 ± 1.42 ^Ab^	-	31.91 ± 1.20 ^Aa^	-	53.29 ± 3.92 ^Ab^	0.97 ± 0.03 ^Ab^	41.85 ± 0.22 ^Ab^	1.51 ± 0.11 ^Aa^	78.09 ± 1.73 ^Ab^	0.12 ± 0.01 ^Aa^
		HE	0.21 ± 0.01 ^Ab^	-	-	-	-	-	-	42.31 ± 1.31 ^Ab^	1.35 ± 0.08 ^Ab^	77.54 ± 1.18 ^Aa^	0.12 ± 0.02 ^Aa^
2019	L1	AD	0.25 ± 0.01 ^Ba^*	54.92 ± 0.50 ^Aa^	0.80 ± 0.03 ^A^	30.83 ± 2.37 ^Aa^	-	73.31 ± 1.06 ^Ba^	0.57 ± 0.01 ^Ba^	49.72 ± 1.10 ^Ba^*	0.96 ± 0.03 ^Ba^	82.53 ± 2.88 ^Ba^*	0.16 ± 0.01 ^Aa^
		HE	0.16 ± 0.01 ^Aa^*	-	-	-	-	-	-	47.46 ± 0.47 ^Ba^*	1.04 ± 0.05 ^Ba^	90.57 ± 0.96 ^Ba^*	0.14 ± 0.01 ^Aa^
	L2	AD	0.21 ± 0.02 ^Aa^*	45.34 ± 5.01 ^Ab^	-	29.21 ± 1.31 ^Aa^	-	64.35 ± 2.66 ^Bb^	0.76 ± 0.01 ^Bb^	46.48 ± 1.96 ^Ba^	1.11 ± 0.04 ^Bb^	90.85 ± 3.32 ^Bb^	0.16 ± 0.02 ^Ba^
		HE	0.17 ± 0.01 ^Ba^*	-	-	-	-	-	-	43.34 ± 2.05 ^Ab^	1.21 ± 0.06 ^Ab^	91.16 ± 1.27 ^Ba^	0.15 ± 0.02 ^Aa^
2020	L1	AD	0.24 ± 0.02 ^Ba^*	50.08 ± 1.88 ^Ba^	0.89 ± 0.04 ^B^	29.96 ± 1.14 ^Aa^	-	72.77 ± 1.19 ^Ba^	0.65 ± 0.01 ^Ca^	45.91 ± 1.25 ^Ba^	1.16 ± 0.07 ^Ca^	87.52 ± 4.16 ^Ba^*	0.13 ± 0.01 ^Ba^
		HE	0.17 ± 0.01 ^Aa^*	-	-	-	-	-	-	43.55 ± 1.11 ^Ba^	1.23 ± 0.08 ^Ca^	96.26 ± 2.94 ^Ca^*	0.11 ± 0.01 ^Ba^
	L2	AD	0.18 ± 0.01 ^Ab^*	39.56 ± 0.60 ^Ab^	-	29.55 ± 2.38 ^Aa^	-	54.34 ± 1.84 ^Ab^	0.91 ± 0.02 ^Cb^	33.98 ± 6.11 ^Ab^	1.79 ± 0.18 ^Ab^	72.55 ± 1.44 ^Cb^	0.12 ± 0.02 ^ABa^
		HE	0.15 ± 0.01 ^Ba^*	-	-	-	-	-	-	33.52 ± 7.20 ^Aa^	1.77 ± 0.26 ^Bb^	80.03 ± 4.71 ^Ab^	0.11 ± 0.01 ^Ba^
Positive control			Trolox (mg/mL) IC_50_ = 0.24 ± 0.01	Rosmarinic acid (45 µg/mL; wo/EDTA) 43.27 ± 3.50% inhibition				Rosmarinic acid (15 µg/mL) 44.43 ± 2.62% inhibition		Rosmarinic acid (120 µg/mL) 95.71 ± 8.55% inhibition		Rosmarinic acid (µg/mL) IC_50_ = 22.05 ± 1.02	

Abbreviations: AD: aqueous decoction and HE: hydroethanolic extractions; Loc.: location; E.M.: extraction method; Tukey’s post hoc test was performed to analyze significant statistical differences between extraction methods (*), between harvest years for the same location (different capital letters), and between locations for the same year (different lowercase letters) if *p* < 0.05. Results are presented as mean ± standard deviation (n = 3). Trolox was used as the positive control for ABTS^•+^ scavenging, and RA was used for all the other assays, please see Section 2.4.

**Table 3:** Assessment of *T. carnosus* extracts neuroprotective, anti-aging, and anti-diabetic potential evaluated as target enzymatic inhibition.

Year	Loc.	E.M.	AChE	Tyrosinase	Elastase	α-Amylase	α-Glucosidase
			% Inhibition	% Inhibition	% Inhibition	% Inhibition	% Inhibition
2018	L1	AD	60.38 ± 6.17 ^ABa^*	18.07 ± 4.63 ^Aa^*	6.3 ± 0.29 ^Aa^	0.23 ± 0.12 ^Aa^*	18.89 ± 3.46 ^Aa^
		HE	44.26 ± 2.56 ^Aa^*	38.33 ± 0.90 ^Aa^*	-	3.74 ± 0.23 ^Aa^*	15.79 ± 0.93 ^Aa^
	L2	AD	46.31 ± 2.56 ^Ab^*	19.11 ± 4.40 ^Aa^*	5.15 ± 3.38 ^Aa^	0.41 ± 0.06 ^Aa^*	12.28 ± 2.36 ^Aa^*
		HE	38.68 ± 1.63 ^Abb^*	33.53 ± 1.40 ^Ab^*	-	3.65 ± 0.16 ^Aa^*	26.64 ± 0.76 ^Ab^*
2019	L1	AD	61.47 ± 1.88 ^Aa^*	26.63 ± 1.63 ^Ba^*	7.32 ± 3.22 ^Aa^	1.49 ± 0.08 ^Ba^*	27.37 ± 2.37 ^Ba^*
		HE	41.98 ± 6.00 ^Aa^*	33.25 ± 2.58 ^Ba^*	-	3.76 ± 0.59 ^Aa^*	12.2 ± 3.34 ^Aa^*
	L2	AD	57.36 ± 2.56 ^Ba^*	20.56 ± 2.36 ^Ab^*	5.56 ± 0.47 ^Aa^	0 ± 0 ^Bb^*	15.44 ± 3.79 ^ABb^*
		HE	31.97 ± 4.65 ^Ab^*	38.33 ± 0.45 ^Bb^*	-	5.46 ± 0.38 ^Bb^*	21.26 ± 1.91 ^Bb^*
2020	L1	AD	54.10 ± 2.56 ^Ba^*	21.55 ± 0.06 ^Aa^*	6.50 ± 1.08 ^Aa^	0.59 ± 0.01 ^Ca^*	19.75 ± 3.76 ^Aa^
		HE	43.03 ± 1.63 ^Aa^*	30.50 ± 0.83 ^Ba^*	-	3.26 ± 0.28 ^Aa^*	18.24 ± 0.01 ^Ba^
	L2	AD	61.06 ± 1.42 ^Bb^*	11.27 ± 1.91 ^Bb^*	1.22 ± 0.57 ^Bb^	0.43 ± 0.08 ^Ab^*	18.41 ± 1.6 ^Ba^
		HE	43.44 ± 2.12 ^Ba^*	42.54 ± 1.46 ^Cb^*	-	3.90 ± 0.11 ^Ab^*	15.77 ± 1.85 ^Ca^
Positive control (% inhibition)			Quercetin (120 µg/mL) 48.61 ± 3.50%	Kojic acid (1 mg/mL) 97.04 ± 1.09%	Quercetin (120 µg/mL) 51.20 ± 7.20%	Acarbose (1 mg/mL) 79.48 ± 3.62%	Acarbose (1 mg/mL) 76.67 ± 1.33%

Abbreviations: AD: aqueous decoction and HE: hydroethanolic extractions; AChE: acetylcholinesterase; Loc.: location; E.M.: extraction method; Tukey’s post hoc test was performed to analyze significant statistical differences between extraction methods (*), between harvest years for the same location (different capital letters), and between locations for the same year (different lowercase letters) if *p* < 0.05. Results are presented as mean ± standard deviation (n = 3). Quercetin was used as a positive control for AChE and elastase assay, kojic acid for tyrosinase assay, and acarbose for α-amylase and α-glucosidase assays, please see Section 2.5.

## Data Availability

The data are contained within the article or Supplementary Materials.

## Supplementary Materials

The following supporting information can be downloaded at: https://www.mdpi.com/article/10.3390/antiox12030668/s1, Table S1: Phytochemical composition of *T. carnosus* hydroethanolic (HE) extracts obtained by HPLC/DAD-ESI/MS^n^ analysis.; Table S2: Phytochemical composition of *T. carnosus* aqueous decoction (AD) extracts obtained by HPLC/DAD-ESI/MS^n^ analysis. Figure S1. Chromatograms of hydroethanolic (A) and aqueous decoction (B) extracts of *T. carnosus* obtained by HPLC-DAD. This are examples of the chromatograms used for phytochemicals’ quantification, obtained at 280 nm for polyphenolic compounds, and the chromatogram used for terpenoids quantification at 210 nm (C) in the hydroethanolic extract. For peak identification please refer to Supplementary Tables S1 and S2. Compounds not identified in these chromatograms were identified in HPLC-ESI-MS^n^ analysis but not in the HPLC-DAD chromatogram used for quantification.; Figure S2. Performance statistics for overall model, predictive components and orthogonal components for the correlation between ABTS^•+^ scavenging and *T. carnosus* extracts’ phytochemicals (A: HE extract; B: AD extract) and respective plots of model’s validation. Sig.: significance. Figure S3: Performance statistics for overall model, predictive components and orthogonal components for the correlation between ^•^OH (A) and NO^•^ (B) scavenging and *T. carnosus* AD extracts’ phytochemicals and respective plots of model’s validation; Figure S4: Performance statistics for overall model, predictive components and orthogonal components for the correlation between β-carotene bleaching inhibition and *T. carnosus* extracts’ phytochemicals (A: HE extract; B: AD extract) and respective plots of model’s validation; Figure S5: Performance statistics for overall model, predictive components and orthogonal components for the correlation between acetylcholinesterase inhibition and *T. carnosus* extracts’ phytochemicals (A: HE extract; B: AD extract) and respective plots of model’s validation; Figure S6: Performance statistics for overall model, predictive components and orthogonal components for the correlation between α-glucosidase inhibition and *T. carnosus* extracts’ phytochemicals (A: HE extract; B: AD extract) and respective plots of model’s validation.
