# Supplementary material for: Phytochemical Composition and Antioxidant, Anti-Acetylcholinesterase, and Anti-α-Glucosidase Activity of Thymus carnosus Extracts: A Three-Year Study on the Impact of Annual Variation and Geographic Location
Source: Antioxidants (Basel). 2023 Mar 8;12(3):668. doi: 10.3390/antiox12030668 (PMC10045533; doi:10.3390/antiox12030668)
Supplement: Supplementary file 1 [file antioxidants-12-00668-s001.zip › antioxidants-2233174-supplementary.pdf]

antioxidants

MDPI

Supplementary Materials

Phytochemical Composition and Antioxidant, Anti-Acetylcholinesterase, and Anti-α-Glucosidase Activity of *Thymus carnosus* Extracts: A Three-Year Study on the Impact of Annual Variation and Geographic Location

Carlos Martins-Gomes [1,2], Jan Steck [3], Judith Keller [3], Mirko Bunzel [3], João A. Santos [1,4], Fernando M. Nunes [2,5] and Amélia M. Silva [1,6,*]

[1] Centre for Research and Technology of Agro-Environmental and Biological Sciences (CITAB), University of Trás-os-Montes and Alto Douro (UTAD), Quinta de Prados, 5001-801 Vila Real, Portugal
[2] Chemistry Research Centre - Vila Real (CQ-VR), UTAD, Quinta de Prados, 5001-801 Vila Real, Portugal
[3] Department of Food Chemistry and Phytochemistry, Institute of Applied Biosciences, Karlsruhe Institute of Technology (KIT), Adenauerring 20a, 76131 Karlsruhe, Germany
[4] Department of Physics, School of Sciences and Technology, UTAD, Quinta de Prados, 5001-801 Vila Real, Portugal
[5] Department of Chemistry, School of Life and Environmental Sciences (ECVA), UTAD, Quinta de Prados, 5001-801 Vila Real, Portugal
[6] Department of Biology and Environment, ECVA, UTAD, Quinta de Prados, 5001-801 Vila Real, Portugal
* Correspondence: amsilva@utad.pt; Tel.: +351-259-350-921

Contents:

Table S1. Phytochemical composition of *T. carnosus* hydroethanolic (HE) extracts obtained by HPLC/DAD-ESI/MS^n analysis

Table S2. Phytochemical composition of *T. carnosus* aqueous decoction (AD) extracts obtained by HPLC/DAD-ESI/MS^n analysis.

Figure S1. Chromatograms of hydroethanolic (A) and aqueous decoction (B) extracts of *T. carnosus* obtained by HPLC-DAD.

Figure S2. Performance statistics for overall model, predictive components and orthogonal components for the correlation between $ABTS^{\bullet+}$ scavenging and *T. carnosus* extracts' phytochemicals (A: HE extract; B: AD extract) and respective plots of model's validation. Sig.: significance.

Figure S3. Performance statistics for overall model, predictive components and orthogonal components for the correlation between $HO^{\bullet}$ (A) and $NO^{\bullet}$ (B) scavenging and *T. carnosus* AD extracts' phytochemicals and respective plots of model's validation. Sig.: significance.

Figure S4. Performance statistics for overall model, predictive components and orthogonal components for the correlation between β-carotene bleaching inhibition and *T. carnosus* extracts' phytochemicals (A: HE extract; B: AD extract) and respective plots of model's validation. Sig.: significance.

Figure S5. Performance statistics for overall model, predictive components and orthogonal components for the correlation between acetylcholinesterase inhibition and *T. carnosus* extracts' phytochemicals (A: HE extract; B: AD extract) and respective plots of model's validation. Sig.: significance.

Figure S6. Performance statistics for overall model, predictive components and orthogonal components for the correlation between α-glucosidase inhibition and *T. carnosus* extracts' phytochemicals (A: HE extract; B: AD extract) and respective plots of model's validation. Sig.: significance.

Table S1. Phytochemical composition of *T. carnosus* hydroethanolic (HE) extracts obtained by HPLC/DAD-ESI/MS^n analysis.

	Compound	R.T. (min)	ESI-MS^2	Quantification (mg/g extract)					
				**2018**		**2019**		**2020**	
				L1	L2	L1	L2	L1	L2
**1**	Apigenin-(6,8)-*C*-diglucoside	22.22±0.07	[593]:575;503;473;383;353	0.49±0.08	0.48±0.15	0.27±0.07	0.37±0.04	0.54±0.05	0.57±0.16
**2**	Hydroxyjasmonic acid-(?)-*O*-hexoside	22.50±0.05	[387]:369;225;207;163	n.q.	n.q.	n.q.	n.q.	n.q.	n.q.
**3**	Caffeic acid	22.63±0.08	[179]:135	1.42±0.18	1.58±0.23	1.41±0.09	1.20±0.09	1.28±0.01	1.23±0.15
**4**	Eriodictyol-(?)-*O*-hexoside isomer 1	23.03±0.01	[449]:287	1.61±0.21	1.89±0.05	0.84±0.01	1.12±0.05	1.59±0.06	1.26±0.1
**5**	Unknown	24.35±0.37	[443]:407;375;307;267;161	n.q.	n.q.	n.q.	n.q.	n.q.	n.q.
**6**	Eriodictyol-(?)-*O*-hexoside isomer 2	25.47±0.06	[449]:287	2.22±0.24	1.14±0.05	1.41±0.15	1.21±0.07	2.41±0.09	1.46±0.13
**7**	Quercetin-(?)-*O*-hexoside	25.71±0.09	[463]:301	14.48±1.48	12.74±2.08	21.21±0.35	12.63±1.51	25.92±0.95	15.14±2.14
**8**	Luteolin-(?)-*O*-hexoside isomer 1	27.22±0.06	[447]:285	0.45±0.07	0.63±0.3	0.37±0.01	0.50±0.03	0.91±0.05	0.63±0.13
**9**	Luteolin-(?)-*O*-hexoside-(?)-O-pentoside	28.14±0.05	[579]:477;285	0.66±0.09	1.45±0.25	0.84±0.03	1.07±0.05	1.15±0.08	1.12±0.2
**10**	Luteolin-(?)-*O*-hexoside isomer 2	29.02±0.04	[447]:285	13.09±1.48	15.84±1.61	14.56±0.70	13.04±0.83	19.63±0.75	16.28±1.62
**11**	Salvianolic acid B/E isomer 1	30.93±0.04	[717]:555;519;475;357	n.q.	n.q.	n.q.	n.q.	n.q.	n.q.
**12**	Salvianolic acid A isomer	31.44±0.08	[493]:383;313;295	15.16±2.29	13.75±1.25	14.55±0.94	15.99±1.12	17.90±0.12	12.83±1.86
**13**	Apigenin-(?)-*O*-hexoside	33.35±0.05	[431]:269	n.q.	n.q.	n.q.	n.q.	n.q.	n.q.
**14**	Acetyl-luteolin-(?)-*O*-hexoside-pentoside	33.53±0.06	[621]:579;561;447;327;285	0.99±0.10	0.90±0.01	1.87±0.06	0.87±0.06	2.09±0.17	1.07±0.28
**15**	Rosmarinic acid	32.62±0.03	[359]:223;179;161	29.44±4.76	19.99±2.01	21.90±1.62	23.24±1.72	26.64±0.12	22.17±2.88
**16**	Salvianolic acid K	34.22±0.03	[555]:537;493;359	9.82±1.52	9.17±0.86	10.76±0.75	11.37±0.79	12.42±0.03	9.10±1.35
**17**	Salvianolic acid B/E isomer 2	34.611±0.04	[717]:555;519;475;357	2.36±0.32	2.55±0.65	3.16±0.18	2.34±0.17	3.49±0.07	2.32±0.42
**18**	Salvianolic acid A	35.18±0.02	[493]:295	n.q.	n.q.	n.q.	n.q.	n.q.	n.q.
**19**	Luteolin-(?)-*O*-hexoside-hexoside isomer 1	35.30±0.07	[609]:447;323;285	1.97±0.25	1.50±0.65	1.31±0.16	1.16±0.06	2.16±0.12	1.29±0.34
**20**	Quercetin-(?)-*O*-hexoside-hexuronide	36.11±0.03	[639]:301	1.83±0.16	1.98±0.69	3.26±0.39	1.69±0.10	2.57±0.20	2.56±0.50
**21**	Luteolin-(?)-*O*-hexoside-hexoside isomer 2	39.83±0.13	[609]:447;323;285	1.33±0.15	1.58±0.43	1.69±0.09	1.27±0.09	1.68±0.24	1.82±0.14
**22**	Oleanolic acid [a]			45.75±5.88	34.85±3.64	48.14±1.88	45.16±4.44	55.80±2.30	41.30±3.22
**23**	Ursolic acid [a]			42.67±4.13	38.58±4.24	40.53±1.54	46.80±4.32	51.84±3.12	40.52±3.45

**Total phenolic compounds**	97.34±13.03	87.17±11.17	99.50±3.80	89.07±6.61	122.36±2.59	90.82±11.58
**Total flavonoids**	39.17±4.09	40.13±6.15	47.71±0.65	34.94±2.83	60.63±2.46	43.20±5.34
**Total phenolic acids**	58.20±9.03	47.01±5.02	51.79±3.51	54.14±3.89	61.73±0.19	47.61±6.53
**Total terpenoids**	88.42±9.99	73.43±7.88	88.67±3.41	91.96±8.75	107.64±5.41	81.82±6.67

RT: retention time; ESI-MS^2 – Fragment ions obtained after fragmentation of the pseudo-molecular ion [M]$^-$; n.q.: detected but not quantified; n.d.: not detected; [a] Identified and quantified by comparison to a commercial standard only. Results are presented as mean ± standard deviation. n = 3.

Table S2. Phytochemical composition of *T. carnosus* aqueous decoction (AD) extracts obtained by HPLC/DAD-ESI/MS^n analysis.

	Compound	R.T. (min)	ESI-MS^2	Quantification (mg/g extract)					
				**2018**		**2019**		**2020**	
				L1	L2	L1	L2	L1	L2
**1**	Apigenin-(6,8)-*C*-diglucoside	22.22±0.07	[593]:575;503;473;383;353	0.53±0.01	0.39±0.04	0.43±0.06	0.44±0.02	0.44±0.08	0.46±0.07
**2**	Hydroxyjasmonic acid-(?)-*O*-hexoside	22.50±0.05	[387]:369;225;207;163	n.q.	n.q.	n.q.	n.q.	n.q.	n.q.
**3**	Caffeic acid	22.63±0.08	[179]:135	3.93±0.38	2.79±0.21	4.10±0.13	3.12±0.19	3.03±0.19	2.74±0.05
**4**	Eriodictyol-(?)-*O*-hexoside isomer 1	23.03±0.01	[449]:287	1.88±0.09	0.95±0.09	1.16±0.09	1.47±0.11	2.19±0.04	1.21±0.07
**5**	Unknown	24.35±0.37	[443]:407;375;307;267;161	n.q.	n.q.	n.q.	n.q.	n.q.	n.q.
**6**	Eriodictyol-(?)-*O*-hexoside isomer 2	25.47±0.06	[449]:287	1.99±0.09	1.02±0.03	1.37±0.07	1.21±0.07	2.26±0.01	1.37±0.07
**7**	Quercetin-(?)-*O*-hexoside	25.71±0.09	[463]:301	6.70±1.38	3.10±0.01	19.48±0.35	3.79±0.38	11.42±0.59	4.38±0.62
**8**	Luteolin-(?)-*O*-hexoside isomer 1	27.22±0.06	[447]:285	0.80±0.13	0.39±0.04	0.69±0.09	0.51±0.01	1.48±0.06	0.74±0.08
**9**	Luteolin-(?)-*O*-hexoside-(?)-O-pentoside	28.14±0.05	[579]:477;285	1.01±0.01	1.07±0.16	1.41±0.16	1.03±0.01	1.54±0.05	0.98±0.07
**10**	Luteolin-(?)-*O*-hexoside isomer 2	29.02±0.04	[447]:285	8.80±1.11	6.28±0.50	11.82±1.08	7.56±0.06	13.82±0.50	8.66±0.74
**11**	Salvianolic acid B/E isomer 1	30.93±0.04	[717]:555;519;475;357	n.q.	n.q.	n.q.	n.q.	n.q.	n.q.
**12**	Salvianolic acid A isomer	31.44±0.08	[493]:383;313;295	8.66±0.89	6.57±0.47	12.24±0.62	8.39±0.30	10.59±0.30	7.52±0.57
**13**	Apigenin-(?)-*O*-hexoside	33.35±0.05	[431]:269	n.q.	n.q.	n.q.	n.q.	n.q.	n.q.
**14**	Acetyl-luteolin-(?)-*O*-hexoside-pentoside	33.53±0.06	[621]:579;561;447;327;285	1.01±0.08	0.54±0.02	2.17±0.24	0.65±0.01	1.66±0.05	0.76±0.04
**15**	Rosmarinic acid	32.62±0.03	[359]:223;179;161	11.00±2.42	4.48±0.89	20.12±0.29	6.67±0.56	10.94±0.63	8.08±1.25
**16**	Salvianolic acid K	34.22±0.03	[555]:537;493;359	8.16±0.53	5.74±0.18	13.80±0.75	7.86±0.41	10.32±0.43	6.70±0.57

**17**	Salvianolic acid B/E isomer 2	34.61±0.04	[717]:555;519;475;357	1.90±0.16	4.15±0.40	2.50±0.10	1.12±0.04	1.39±0.02	1.36±0.12
**18**	Salvianolic acid A	35.18±0.02	[493]:295	n.q.	n.q.	n.q.	n.q.	n.q.	n.q.
**19**	Luteolin-(?)-*O*-hexoside-hexoside isomer 1	35.30±0.07	[609]:447;323;285	2.74±0.09	1.58±0.26	2.08±0.22	1.61±0.04	2.85±0.23	1.48±0.07
**20**	Quercetin-(?)-*O*-hexoside-hexuronide	36.11±0.03	[639]:301	1.60±0.13	1.12±0.09	3.99±0.39	1.33±0.01	2.26±0.22	1.61±0.14
**21**	Luteolin-(?)-*O*-hexoside-hexoside isomer 2	39.83±0.13	[609]:447;323;285	1.07±0.07	0.82±0.06	1.94±0.25	0.98±0.07	1.33±0.21	1.06±0.06
**22**	Oleanolic acid [a]			n.d.	n.d.	n.d.	n.d.	n.d.	n.d.
**23**	Ursolic acid [a]			n.d.	n.d.	n.d.	n.d.	n.d.	n.d.
			**Total phenolic compounds**	61.78±6.28	37.96±1.15	99.86±4.13	48.02±0.15	78.63±3.25	48.86±4.75
			**Total flavonoids**	28.14±2.65	17.24±0.12	45.69±2.10	20.58±0.17	41.24±1.88	23.76±0.86
			**Total phenolic acids**	33.65±3.62	20.70±1.27	54.17±2.03	27.43±0.32	37.39±1.62	26.40±2.44
			**Total terpenoids**	n.d.	n.d.	n.d.	n.d.	n.d.	n.d.

RT: retention time; ESI-MS^2 – Fragment ions obtained after fragmentation of the pseudo-molecular ion $[M]^-$; n.q.: detected but not quantified; n.d.: not detected; [a] Identified and quantified by comparison to a commercial standard only. Results are presented as mean ± standard deviation. n = 3.

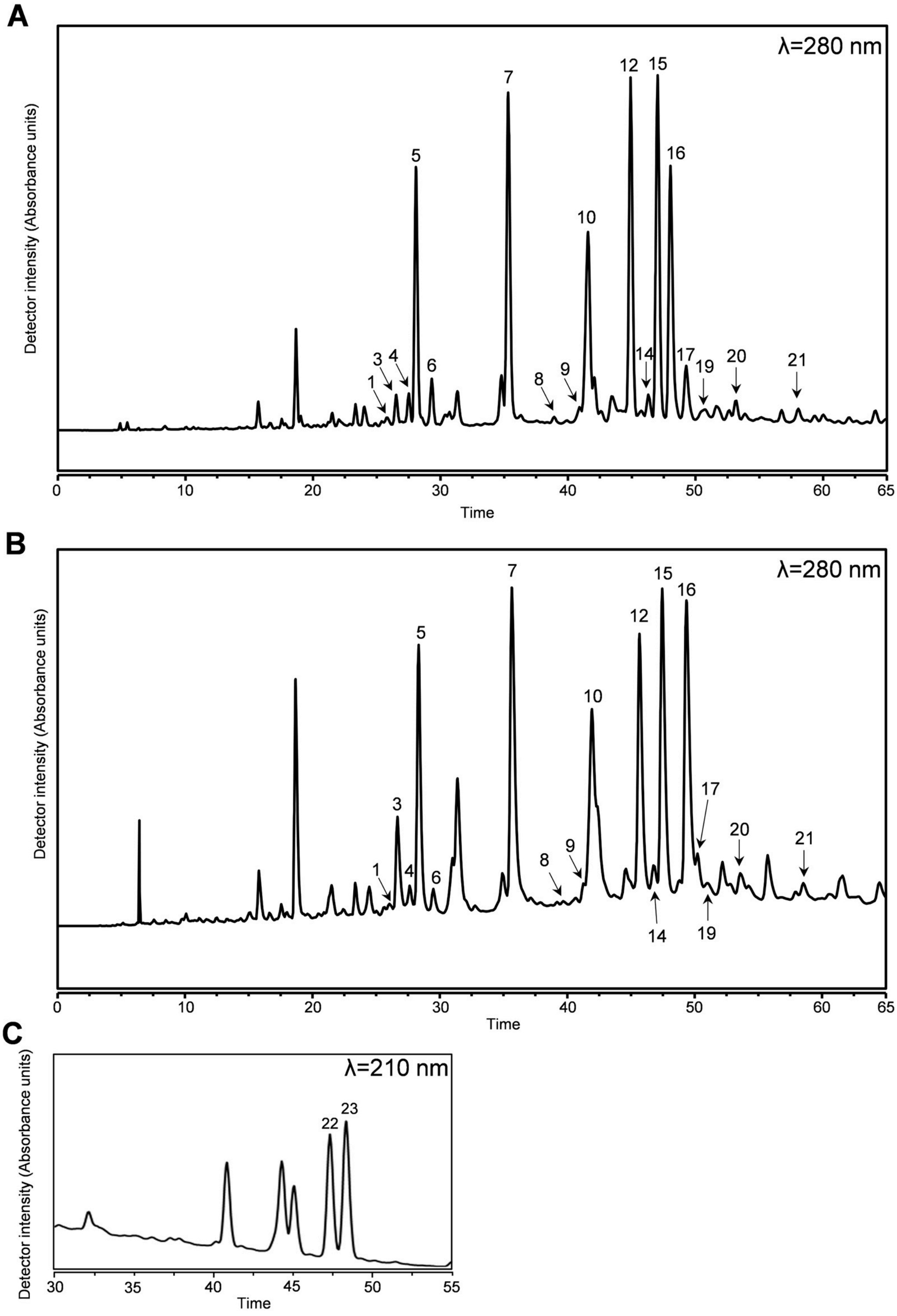

Figure S1. Chromatograms of hydroethanolic (A) and aqueous decoction (B) extracts of *T. carnosus* obtained by HPLC-DAD. This are examples of the chromatograms used for phytochemicals' quantification, obtained at 280 nm for polyphenolic compounds, and the chromatogram used for terpenoids quantification at 210 nm (C) in the hydroethanolic extract. For peak identification please refer to Supplementary tables S1 and S2. Compounds not identified in these chromatograms were identified in HPLC-ESI-MS^n analysis but not in the HPLC-DAD chromatogram used for quantification.

A

Component	R2X	R2X (cum)	Eigenvalue	R2	R2 (cum)	Q2	Limit	Q2 (cum)	R2Y	R2Y (cum)	Eigenvalue Y	Sig.
Model		0,648			0,886			0,647		1		
Predictive		0,282			0,886			0,647		1		
P1	0,282	0,282	5,07	0,886	0,886	0,647	0,01	0,647	1	1	1	R1
Orthogonal in X(OPLS)		0,366			0							
O1	0,266	0,266	4,8	0	0							R1
O2	0,0997	0,366	1,79	0	0							R1

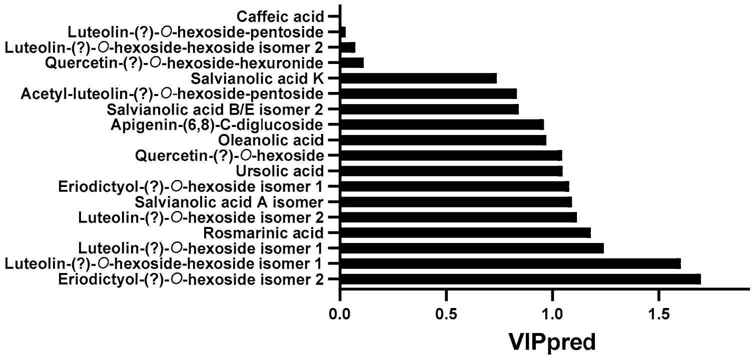

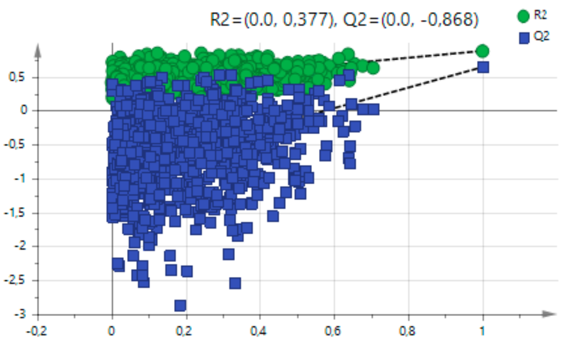

B

Component	R2X	R2X (cum)	Eigenvalue	R2	R2 (cum)	Q2	Limit	Q2 (cum)	R2Y	R2Y (cum)	Eigenvalue Y	Sig.
Model		0,643			0,874			0,847		1		
Predictive		0,643			0,874			0,847		1		
P1	0,643	0,643	10,3	0,874	0,874	0,847	0,01	0,847	1	1	1	R1

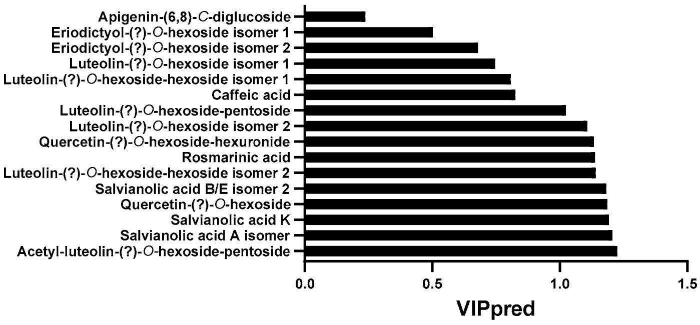

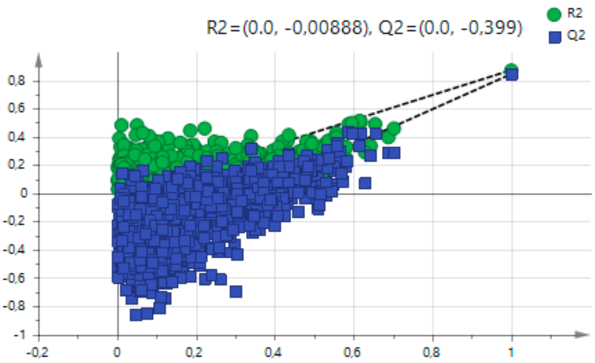

Figure S2. Performance statistics for overall model, predictive components and orthogonal components for the correlation between ABTS$^{\bullet+}$ scavenging and *T. carnosus* extracts' phytochemicals (A: HE extract; B: AD extract) and respective plots of model's validation. Sig.: significance.

A

Component	R2X	R2X (cum)	Eigenvalue	R2	R2 (cum)	Q2	Limit	Q2 (cum)	R2Y	R2Y (cum)	Eigenvalue Y	Sig.
Model		0,93			0,871			0,795		1		
Predictive		0,473			0,871			0,795		1		
P1	0,473	0,473	7,57	0,87	0,871	0,79	0,01	0,795	1	1	1	R1
Orthogonal in X(OPLS)		0,457			0							
O1	0,271	0,271	4,33	0	0							R1
O2	0,186	0,457	2,98	0	0							R1

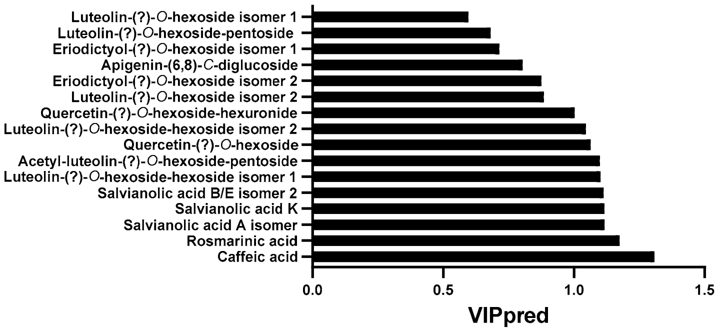

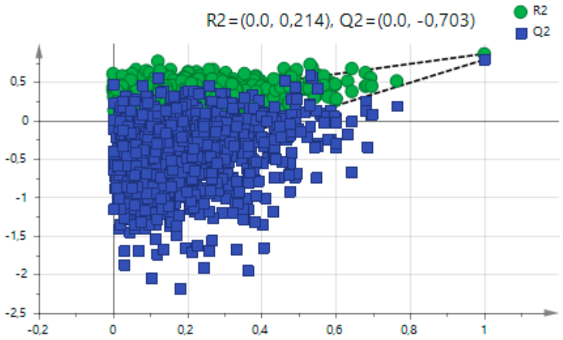

B

Component	R2X	R2X (cum)	Eigenvalue	R2	R2 (cum)	Q2	Limit	Q2 (cum)	R2Y	R2Y (cum)	Eigenvalue Y	Sig.
Model		0,642			0,787			0,773		1		
Predictive		0,642			0,787			0,773		1		
P1	0,642	0,642	10,3	0,787	0,787	0,773	0,01	0,773	1	1	1	R1

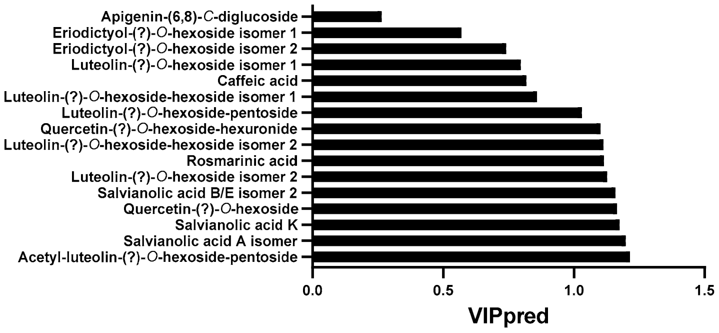

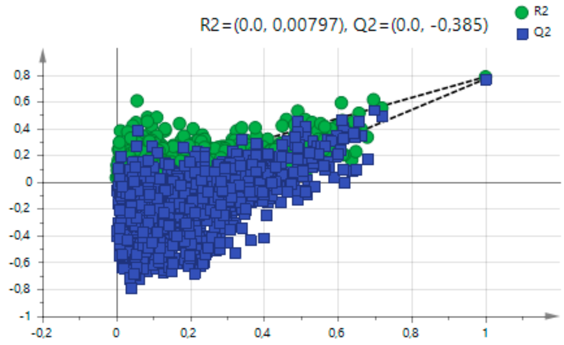

Figure S3. Performance statistics for overall model, predictive components and orthogonal components for the correlation between $HO^{\bullet}$ (A) and $NO^{\bullet}$ (B) scavenging and *T. carnosus* AD extracts' phytochemicals and respective plots of model's validation. Sig.: significance.

A

Component	R2X	R2X (cum)	Eigenvalue	R2	R2 (cum)	Q2	Limit	Q2 (cum)	R2Y	R2Y (cum)	Eigenvalue Y	Sig.
Model		0,587			0,877			0,773		1	0,587	
Predictive		0,289			0,877			0,773		1		
P1	0,289	0,289	5,2	0,87	0,877	0,77	0,01	0,773	1	1	1	R1
Orthogonal in X(OPLS)		0,298			0							
O1	0,298	0,298	5,36	0	0							R1

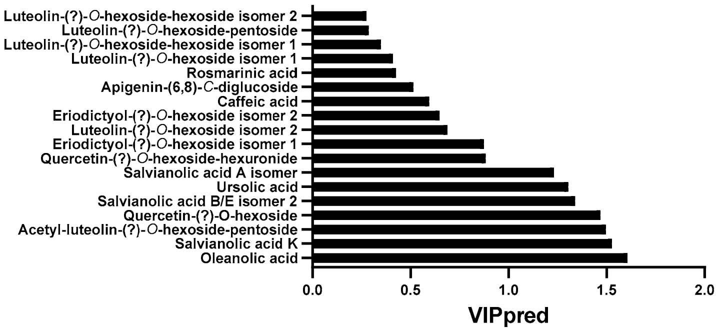

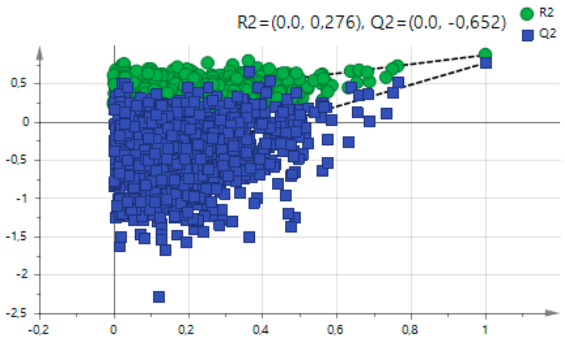

B

Component	R2X	R2X (cum)	Eigenvalue	R2	R2 (cum)	Q2	Limit	Q2 (cum)	R2Y	R2Y (cum)	Eigenvalue Y	Sig.
Model		0,978			0,887			0,665		1		
Predictive		0,066			0,887			0,665		1	0,066	
P1	0,066	0,066	1,06	0,887	0,887	0,665	0,01	0,665	1	1	1	R1
Orthogonal in X(OPLS)		0,912			0							
O1	0,592	0,592	9,47	0	0							R1
O2	0,151	0,743	2,42	0	0							R1
O3	0,138	0,881	2,21	0	0							R1
O4	0,0312	0,912	0,499	0	0							R1

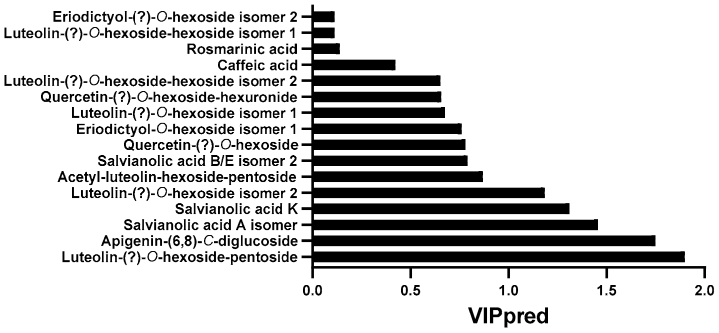

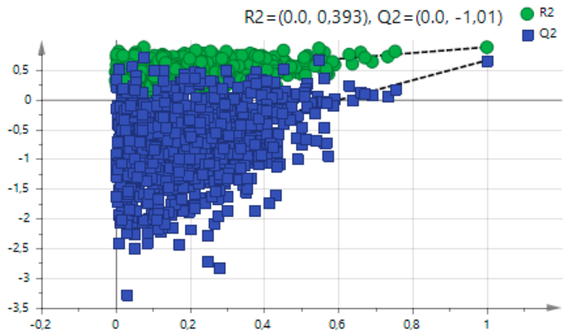

Figure S4. Performance statistics for overall model, predictive components and orthogonal components for the correlation between β-carotene bleaching inhibition and *T. carnosus* extracts' phytochemicals (A: HE extract; B: AD extract) and respective plots of model's validation. Sig.: significance.

A

Component	R2X	R2X (cum)	Eigenvalue	R2	R2 (cum)	Q2	Limit	Q2 (cum)	R2Y	R2Y (cum)	Eigenvalue Y	Sig.
Model		0,592			0,715			0,23		1	0,592	
Predictive		0,133			0,715			0,23		1	0,133	
P1	0,133	0,133	2,39	0,71	0,715	0,23	0,01	0,23	1	1	1	R1
Orthogonal in X(OPLS)		0,459			0							
O1	0,364	0,364	6,56	0	0							NS
O2	0,094	0,459	1,7	0	0							R1

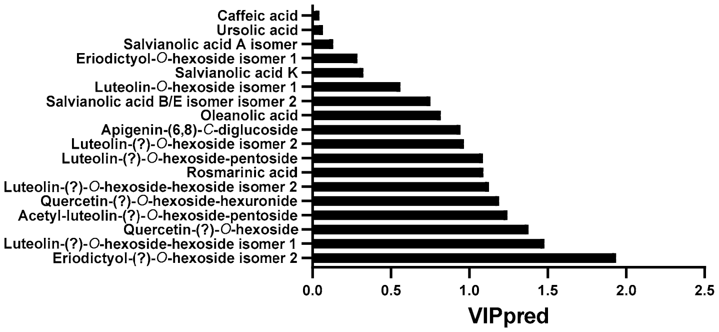

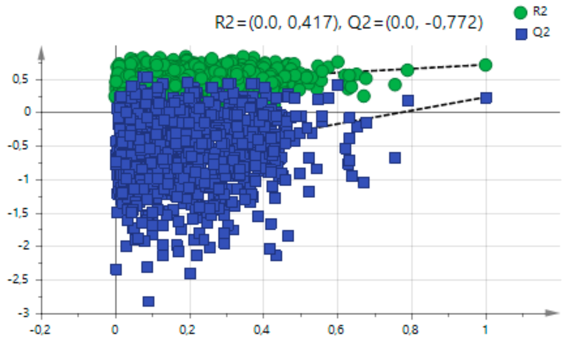

B

Component	R2X	R2X (cum)	Eigenvalue	R2	R2 (cum)	Q2	Limit	Q2 (cum)	R2Y	R2Y (cum)	Eigenvalue Y	Sig.
Model		0,981			0,825			0,517		1		
Predictive		0,12			0,825			0,517		1	0,12	
P1	0,12	0,12	1,92	0,825	0,825	0,517	0,01	0,517	1	1	1	R1
Orthogonal in X(OPLS)		0,861			0							
O1	0,6	0,6	9,61	0	0							R1
O2	0,165	0,765	2,64	0	0							R1
O3	0,0686	0,834	1,1	0	0							R1
O4	0,0273	0,861	0,437	0	0							R1

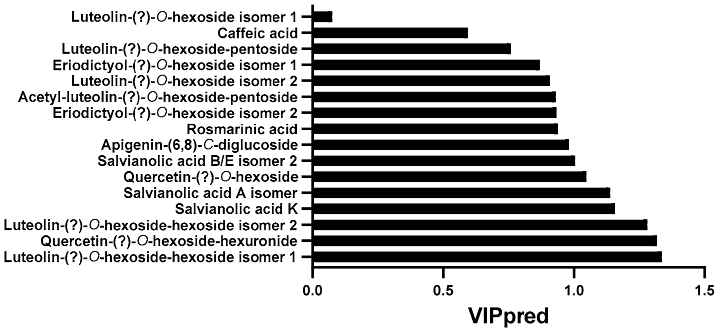

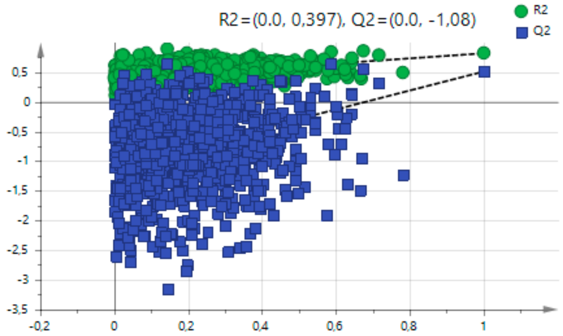

Figure S5. Performance statistics for overall model, predictive components and orthogonal components for the correlation between acetylcholinesterase inhibition and *T. carnosus* extracts' phytochemicals (A: HE extract; B: AD extract) and respective plots of model's validation. Sig.: significance.

A

Component	R2X	R2X (cum)	Eigenvalue	R2	R2 (cum)	Q2	Limit	Q2 (cum)	R2Y	R2Y (cum)	Eigenvalue Y	Sig.
Model		0,756			0,924			0,744		1		
Predictive		0,143			0,924			0,744		1		
P1	0,143	0,143	2,57	0,92	0,924	0,74	0,01	0,744	1	1	1	R1
Orthogonal in X(OPLS)		0,614			0							
O1	0,404	0,404	7,27	0	0							R1
O2	0,133	0,537	2,39	0	0							R1
O3	0,077 2	0,614	1,39	0	0							R1

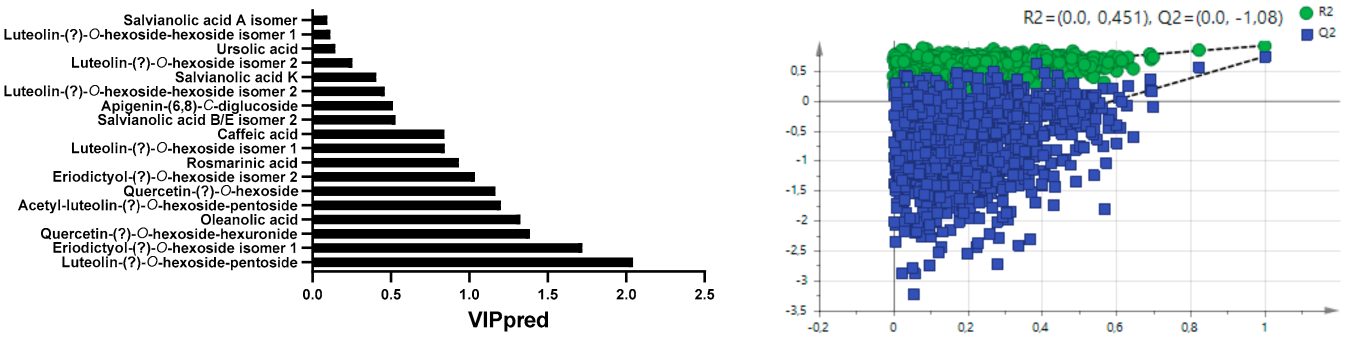

B

Compo-nent	R2X	R2X (cum)	Eigenvalue	R2	R2 (cum)	Q2	Limit	Q2 (cum)	R2Y	R2Y (cum)	Eigen-value Y	Sig.
Model		0.639			0.728			0.653		1		
Predictive		0.639			0.728			0.653		1		
P1	0.639	0.639	10.2	0,728	0,728	0,653	0,01	0,653	1	1	1	R1

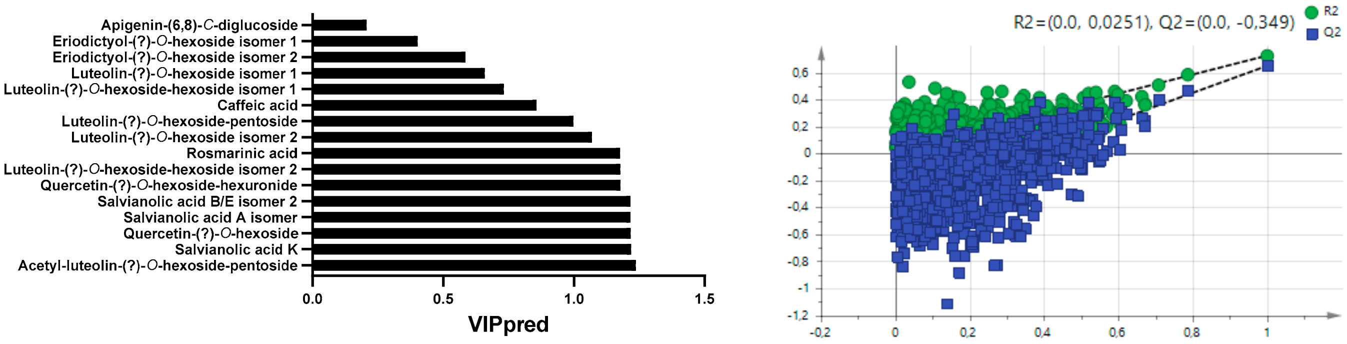

Figure S6. Performance statistics for overall model, predictive components and orthogonal components for the correlation between α-glucosidase inhibition and *T. carnosus* extracts' phytochemicals (A: HE extract; B: AD extract) and respective plots of model's validation. Sig.: significance.
